# PET-CT-guided, symptom-based, patient-initiated surveillance versus clinical follow-up in head neck cancer patients (PETNECK2): study protocol for a multicentre feasibility study and non-inferiority, randomised, phase III trial

**DOI:** 10.1186/s12885-024-12470-9

**Published:** 2024-07-10

**Authors:** Paul Nankivell, Piers Gaunt, Claire Gaunt, Julia Sissons, Evaggelia Liaskou, Yolande Jefferson, Tessa Fulton-Lieuw, Saloni Mittal, Hisham Mehanna, PETNECK2 Research Team, Ahmad Abou-Foul, Andreas Karwath, Ava Lorenc, Barry Main, Colin Greaves, David Moore, Denis Secher, Eila Watson, Georgios Gkoutos, Gozde Ozakinci, Jane Wolstenholme, Janine Dretzke, Jo Brett, Joan Duda, Lauren Matheson, Marcus Jepson, Mary Wells, Melanie Calvert, Pat Rhodes, Philip Kiely, Steve Thomas, Stuart Winter, Wai-lup Wong

**Affiliations:** 1 Institute of Head and Neck Studies and Education (InHANSE), Institute of Cancer and Genomic Sciences, University of Birmingham, Birmingham, B15 2TT UK; 2 Cancer Research UK Clinical Trials Unit (CRCTU), University of Birmingham, Birmingham, Edgbaston, Birmingham. B15 2TT, UK

**Keywords:** Feasibility study, Phase III randomised clinical trial, Head and neck cancer, Patient-initiated follow-up

## Abstract

**Background:**

Approximately 40% of treated head and neck cancer (HNC) patients develop recurrence. The risk of recurrence declines with time from treatment. Current guidelines recommend clinical follow-up every two months for the first two years after treatment, with reducing intensity over the next three years. However, evidence for the effectiveness of these regimes in detecting recurrence is lacking, with calls for more flexible, patient-centred follow-up strategies.

**Methods:**

PETNECK2 is a UK-based multi-centre programme examining a new paradigm of follow-up, using positron emission tomography-computed tomography (PET-CT)-guided, symptom-based, patient-initiated surveillance. This paradigm is being tested in a unblinded, non-inferiority, phase III, randomised controlled trial (RCT). Patients with HNC, one year after completing curative intent treatment, with no clinical symptoms or signs of loco-regional or distant metastasis will be randomised using a 1:1 allocation ratio to either regular scheduled follow-up, or to PET-CT guided, patient-initiated follow-up. Patients at a low risk of recurrence (negative PET-CT) will receive a face-to-face education session along with an Information and Support (I&S) resource package to monitor symptoms and be in control of initiating an urgent appointment when required. The primary outcome of the RCT is overall survival. The RCT also has an in-built pilot, a nested QuinteT Recruitment Intervention (QRI), and a nested mixed-methods study on patient experience and fear of cancer recurrence (FCR). An initial, single-arm feasibility study has been completed which determined the acceptability of the patient-initiated surveillance intervention, the completion rates of baseline questionnaires, and optimised the I&S resource prior to implementation in the RCT.

**Discussion:**

We hypothesise that combining an additional 12-month post-treatment PET-CT scan and I&S resource will both identify patients with asymptomatic recurrence and identify those at low risk of future recurrence who will be empowered to monitor their symptoms and seek early clinical follow-up when recurrence is suspected. This change to a patient-centred model of care may have effects on both quality of life and fear of cancer recurrence.

**Trial registration:**

ISRCTN: 13,709,798; 15-Oct-2021.

**Supplementary Information:**

The online version contains supplementary material available at 10.1186/s12885-024-12470-9.

## Background

Head and neck cancer (HNC) is the sixth most common cancer worldwide, with 640,000 cases diagnosed annually [1]. There are 12,000 new cases diagnosed and > 4000 deaths annually in the United Kingdom [2]. Approximately 40% of patients (range 20–57%) treated for HNC commonly develop cancer recurrence at the primary site or the draining lymph nodes [3]. The majority occur in the first two years after treatment (62% in first year, 82% within two years) [4–6]. Rates of distant metastases are reported to be between 10 and 20% [7, 8]. Despite significant morbidity, salvage surgery provides the best opportunity for long-term survival in patients with recurrent HNC, but this is only possible if the recurrent disease is amenable to resection, which is more likely to be the case with early detection [9, 10].

The most recent UK national head and neck cancer guidelines recommend that patients undergo clinical follow-up every two months for the first two years after treatment, and then every three-to-six months for the next three years [11]. Regular follow-up with clinical examination for a minimum of five years following treatment end is also a standard recommendation from almost all international head and neck cancer societies.

Despite being the international standard, current strategies for HNC surveillance lack a robust evidence base for their effectiveness or efficiency and are inflexible. The rate of cancer recurrence detection in asymptomatic patients through routine clinical surveillance is low [12], with published data suggesting only between 27 and 51% of patients with recurrence can be offered salvage treatment [13, 14]. Importantly, patients are calling for more flexible, patient-centred follow-up [15], with HNC patients reporting current follow-up regimes being too frequent, favouring intensive follow-up in the first year only, with less intensive, symptom-based appointments thereafter [16]. However, this is balanced by HNC patients’ concern that their cancer may return or progress [17, 18]. This fear of cancer recurrence (FCR) has a major impact on patients’ overall quality of life [19, 20] and is well documented in HNC patients [18, 20–22], yet no studies have examined the effect of different follow-up regimens on FCR. Furthermore, this is coupled with limited, poor quality, retrospective and conflicting evidence regarding the relative efficacy of the current strategy of routine follow-up versus symptom-driven self-referral strategies in clinical HNC management: four studies report a difference in overall survival between routine follow-up and self-referral for HNC patients [23–28]. Adding to this issue is the dramatic and exponential rise in the incidence of oropharyngeal squamous cell carcinoma (OPC) driven by human papillomavirus (HPV) infection [29–31], which has higher survival rates [2]. Taken together this contributes to a rapidly enlarging cohort of cancer survivors requiring follow-up, placing significant pressure on finite health care resources [32].

As demonstrated by the PET-NECK trial, positron emission tomography-computed tomography (PET-CT) scans at three months post chemoradiotherapy are an effective strategy to select patients for ongoing management - avoiding a routine neck dissection (the previous standard of care) in > 80% of patients, with a reduction in harm, and saving of £1,415 per patient to the UK’s National Health Service (NHS) [33, 34]. This has become the standard of care in most countries, and we aim in PETNECK2 to develop and test the efficacy and cost-effectiveness of a better paradigm for post-treatment surveillance for patients with HNC.

PETNECK2 will examine the effectiveness of PET-CT imaging - introduced at one year after completion of treatment – to determine patients at low risk of recurrence. We hypothesise that this PET-CT scan may identify some patients with asymptomatic recurrence (allowing earlier intervention with salvage therapies), but also those at very low risk of future recurrence. These low-risk patients would then receive a face-to-face education session along with an Information and Support (I&S) resource package, which includes information regarding symptom monitoring, and how to initiate an urgent appointment if they have any concerns or recurrence is suspected. Therefore, instead of regular scheduled follow-up appointments, patients would be in control of when they return for follow-up visits (patient-initiated follow-up).

This programme of work included development of a bespoke I&S resource package [35–37] and an initial feasibility study to optimise the education session and I&S resource package given prior to the phase III Randomised Controlled Trial (RCT), which will formally assess the efficacy and cost-effectiveness of this patient-initiated active surveillance strategy compared to the current standard of care routine scheduled clinical follow-up.

## Design

### Study design

### Feasibility study

**Fig. 1:**
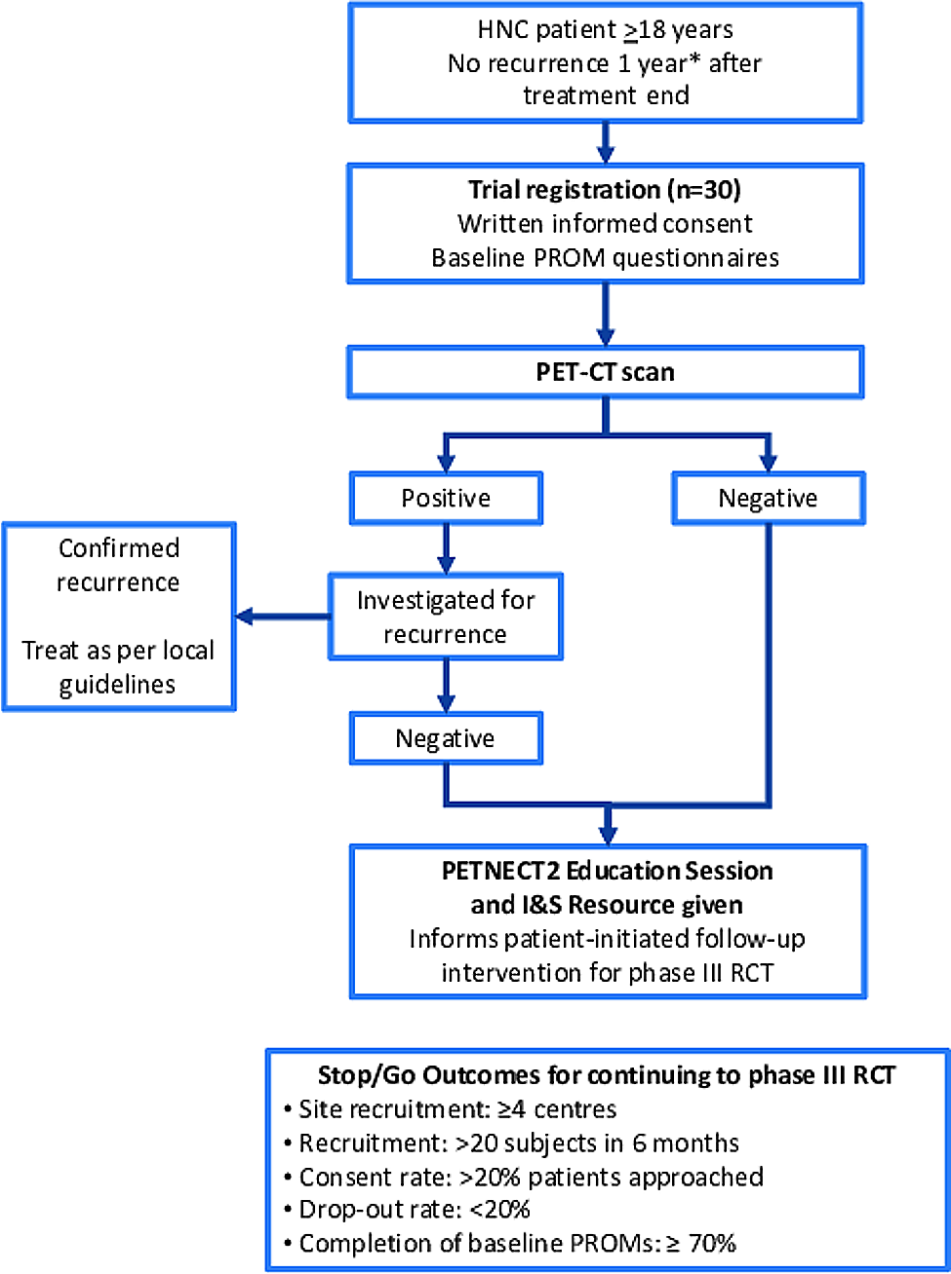
PETNECK2 feasibility study schema. Schema for the initial PETNECK2 feasibility study, which assessed acceptability of the patient-initiated active surveillance strategy to patients and clinicians and defined the intervention for the randomised controlled trial. * 11-to-14 months post-treatment is permitted. HNC, head and neck cancer; I&S, information and support; PROM, patient-reported outcome measures; RCT, randomised controlled trial

A single arm, UK-wide multi-centre, prospective study of 30 patients and their recruiting clinicians to assess the acceptability of the patient-initiated active surveillance to patients and clinicians, and to amend the intervention as required (Fig. 1). This study defined the patient-initiated follow-up strategy that is now being examined within the RCT [38]. Recruitment was over seven months (February to August 2022). Participants were followed up for a minimum of two months. The feasibility study completed on 03-Apr-2023, when all patients reverted to standard of care follow-up.

#### Randomised controlled trial (RCT)

**Fig. 2:**
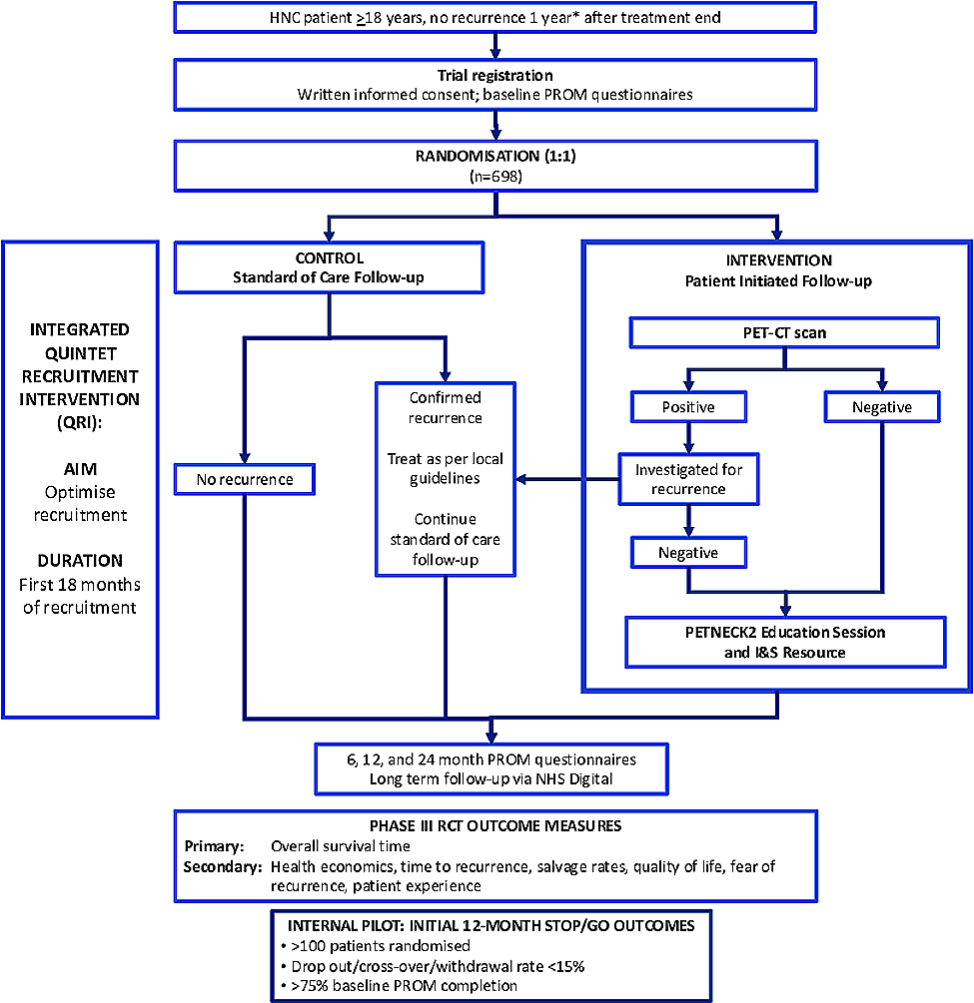
PETNECK2 randomised controlled trial schema. Schema for the PETNECK2 randomised controlled trial to assess the efficacy and cost-effectiveness of patient-initiated active surveillance compared to the routine clinical follow-up. * 6-to-14 months post-treatment is permitted for registration into the RCT. Randomisation must occur 11–14 months post-treatment. HNC, head and neck cancer; I&S, information and support; PROM, patient-reported outcome measures; RCT, randomised controlled trial

An unblinded, UK-wide multicentre, non-inferiority, phase III, RCT to assess the efficacy and cost-effectiveness of patient-initiated active surveillance compared to the routine clinical follow-up (Fig. 2). The trial has an in-built pilot, a nested QuinteT recruitment intervention (QRI), and a nested mixed methods study on patient experience and Fear of Cancer Recurrence (FCR).

Randomisation of 698 patients is performed electronically with a 1:1 allocation ratio and remotely by recruiting centres, using a computer minimisation program incorporating a random element developed by the Cancer Research UK Clinical Trials Unit (CRCTU), Birmingham, UK. Stratification variables are: centre; HPV status/tumour site combination; tumour stage; and baseline FCR score.

Recruitment will be over 33 months, with participants followed up for at least 12 months from randomisation, incorporating follow-up using electronic medical records.

A list of the PETNECK2 centres can be requested from the PETNECK2 Trial Office (PETNECK2@trials.bham.ac.uk). The Standard Protocol Items: Recommendations for Intervention Trials (SPIRIT) checklist is provided as Additional File 1 [39]. The World Health Organization (WHO) Trial Registration Data Set is provided in Additional File 2.

### Patient and public involvement

Work package 1 spans the entire duration of the programme and involves a Patient Advisory Group (PAG). This group consisting of 9 patients and carers, has co-developed the information and support resources (App and booklet) that support the trial intervention. They have reviewed and helped refine the protocol, participant-facing documents, training material for healthcare professionals delivering the study, and provided input into the statistical design of the study, particularly regarding selection of the pre-specified non-inferiority margin. Bespoke training workshops were held for members of the PAG. Representatives of the PAG sit on both the Programme Management Group (PMG) and the Trial Management Group (TMG). They will continue to assess study conduct, will be involved in review of any amendments, and will support dissemination of the study results through existing advocacy activities and through social media channels.

### Patient selection

Patients who have histological or cytological confirmation of oral, oropharyngeal, nasopharyngeal, laryngeal or hypopharyngeal squamous cell carcinoma, are aged ≥ 18 years, are at least 11- to 14-months post completion of curative intent treatment by any modality (surgery, radiation or combination treatments), with no clinical symptoms or signs of loco-regional or distant metastasis and are able to provide written informed consent are eligible for randomisation into the PETNECK2 study. Patients with non-squamous cell carcinoma tumours, or those from sites other than those stated above, who are pregnant, have clinical symptoms or signs of loco-regional or distant metastasis, or are already enrolled in a head and neck cancer clinical trial where scheduled follow-up is a requirement of that protocol are not eligible. For those patients enrolled in the RCT, patient with head and neck cancer of unknown primary are also ineligible. Patients will be recruited from head and neck cancer units based in secondary and tertiary care settings.

For the RCT only, participant registration is permitted from 6-months post completion of curative intent treatment. This aims to increase potential recruitment by identifying participants early and introducing them to the trial. Randomisation can only be performed between 11- and 14-months post completion of curative intent treatment.

### Screening and consent

It is the responsibility of the investigator to obtain written informed consent for each patient prior to performing any trial related procedure in compliance with national regulations (ICH-GCP). Patient information sheets (PIS) are provided to facilitate this process. The site investigator or an appropriately qualified and GCP-trained research nurse, clinical research practitioner, research fellow or NIHR associate PI may obtain written informed consent. PETNECK2 has feasibility- and RCT-specific informed consent forms and PIS, which have been included in Additional Files 3 and 4, respectively.

### Intervention

#### Feasibility study and comparator arm of randomised controlled trial

A PET-CT scan is offered to patients at approximately 12 months following completion of treatment (within 11–14 months). Clinical, radiological and pathological staging is performed according to the UICC TNM Classification of Malignant Tumours staging manual 8th edition [40].

If the PET-CT scan is positive or equivocal for head and neck cancer recurrence, patients will undergo further investigations (as per UK guidelines) to confirm or exclude recurrence (this may include undergoing additional scans, biopsies or examination under anaesthetic). If recurrent cancer is confirmed, they will be treated according to the recommendations of the local multidisciplinary team. Any patient with recurrent cancer should receive scheduled follow-up appointments arranged by their treating clinical team.

If the PET-CT scan is positive but suggests a pathology not thought to represent a HNC recurrence (e.g. a new non-head and neck cancer, or metabolic activity from a non-oncogenic process), further investigations will be offered to the patient guided by the local clinical teams, and a management plan for the patient will be decided based on national guidelines. The patient will still be able to continue with the intervention arm if they wish to.

If the PET-CT scan is negative (with no signs of recurrence or metastases), patients receive an education session delivered by a clinical nurse specialist or Allied Health Professional (APH) (approximately 20–30 min in length) to discuss, demonstrate, and ensure familiarity with the I&S resource.

#### Control arm of randomised controlled trial

Standard of care clinical follow-up as per UK HNC guidelines is followed at the discretion of the participating centre, e.g. two-monthly in the second year after the end of treatment, and three-to-six monthly in years three-to-five.

The schedule of events for patients in either the PETNECK2 feasibility study and RCT have been included in Tables 1 and 2, respectively.

**Table 1:** PETNECK2 feasibility study patient pathway

Assessments	6–14 months post- treatment	Baseline 11–14 months post-treatment	Post PET-CT scan	Follow-up appointment (1–2 months post education session)	Open urgent appointment^a^
Informed consent	x				
Registration	x				
Quality of Life & Resource Use Questionnaires^b^		x		x	
PET-CT scan^c^		x			
PET-CT consultation			x		
PETNECK2 Education Session			x		
Qualitative interview				x	
Clinical Interaction for Symptom Assessment and evaluation					x

^**a**^Multiple and at any time point after the education session for the duration of the feasibility study

^b^To include EQ-5D-5L, EORTC QLQ-C30 and H&N43, Fear of Cancer Recurrence

^c^If recurrent disease diagnosed on PET-CT and confirmed on further investigation, no further study intervention will be undertaken. Follow-up data will still be collected

**Table 2:** PETNECK2 randomised controlled trial patient pathway

Assessments	6–14 months post-treatment	Baseline 11–14 months post- treatment	Post PET-CT scan	Open urgent appointment^d^	6 months* follow-up	12 months* follow-up	24 months* follow-up
Informed consent	x						
Registration	x						
Randomisation		x					
Fear of Cancer Recurrence (FCR) questionnaire		x				x	x
EORTC questionnaires (QLQ-C30 and QLQ-H&N43)		x				x	x
EQ-5D-5L & Resource Use questionnaires		x			x	x	x
Self-efficacy & motivation questionnaire		x			x	x	
CQC question					x	x	x
Blood, oral rinse, and tissue collection^b^		x				x	
Fear of Cancer Recurrence interview^c^		x				x	
PET-CT scan^a^		x					
PET-CT consultation^a^			x				
PETNECK2 Education Session^a^			x				
Clinical interaction for sympton assessment and evaluation				x			

^a^Patients randomised to intervention arm only

^b^Optional - patients consenting to PETNECK2 Collect sub-study

^c^Optional - a subset of patients with clinically significant FCR (taken from baseline FCR questionnaire with cut-off ≥ 22) consenting to FCR interviews. The first of these should take place before the PET-CT scan

^d^As required and at any time point after the education session for the duration of the trial

* Follow-up post-randomisation

Grey boxes indicate assessments completed by the patients but not conducted at sites

### PETNECK2 patient-based follow-up education session

This education session is formed of two parts:

- Before consultation with a nurse/Allied Health Professional (AHP), patients receive the I&S resource as either a paper-based booklet or as a digital resource (web-based or downloadable mobile App). Instructions on how to access the mobile App, unique access codes per patient, and a link to a training video demonstrating a walk-through of the resource features is shared.
- A 20-to-30 min session delivered by a trained nurse/AHP who reviews the I&S resource with the patient, where they explore and address any potential barriers to patient-initiated follow-up and provide written or online information (based on patient preference) containing the following information:
  - Symptoms that necessitate immediate contact with the clinical team.
  - Management of treatment side-effects, e.g. dry mouth, stiffness, difficulty swallowing.
  - How to monitor symptoms by completing the key symptom checker tool in the paper booklet or on the web-based or mobile App.
  - How to use the App and website.
  - Review the contact details of the patient’s local head and neck cancer specialist team, including the patient’s key healthcare worker, and how to contact them (number inputted into the mobile App or booklet).
  - How to initiate an urgent review appointment at any time where, if deemed necessary by the clinical team, they will be guaranteed to be seen in the normal head and neck follow-up clinic within two weeks of contacting the team.

### Patient reported outcome measures

#### Quality of life

Quality of life is assessed using EORTC QLQ-C30 (version 3) to assess global health scale and general patient function and symptoms [41] with the HNC-specific EORTC QLQ-H&N43 module [42], and EQ-5D-5L [43] to assess both disease burden and cost of medical interventions received at baseline (11–14 months post-treatment) for all patients enrolled in the RCT, prior to randomisation, 6 months (EQ-5D-5L only), 12 and 24 months post-randomisation.

#### Resource use

Patients enrolled in the RCT will be asked to complete a non-validated resource use questionnaire that asks about primary care consultations, out of pocket expenses, social care contacts, and employment status. This questionnaire will be required for the analysis of health economics in the RCT. This is completed at baseline (11–14 months post-treatment) prior to randomisation and then at 6-, 12- and 24-months post-randomisation.

#### Self-efficacy and motivation

Patients are asked to answer a set of questions about their self-checking habits. At baseline this ascertains whether they self-check or not, while at 6- and 12-months, it will assess the frequency of their self-checking. Patients are also requested to indicate their confidence levels to know what is normal for them/notice any changes or new symptoms, manage to check monthly for changes, and be able to report when they have noticed a change. This is based on published guidelines for constructing self-efficacy scales [44]. Patients are also asked to complete a modified version of the Treatment Self-Regulation Questionnaire to assess reasons for engaging in patient-initiated follow-up [45]. All patients are asked to complete these questionnaires at baseline, 6- and 12-months post-randomisation. At 6- and 12-months, two items from the Autonomy and Competence in Technology Adoption Questionnaire (ACTA) [46] are also employed to assess the extent to which intervention arm patients perceive that they are competent to use the App/booklet. The self-checking, self-efficacy and motivation questions are included as part of the Questionnaire Pack.

#### Fear of cancer recurrence

A nine-item FCR Inventory-Short form [47] regarding “fear, worry, or concern about cancer returning or progressing” [19] is being used during PETNECK2. This is given to all patients at baseline (11–14 months post-treatment) and at approximately 12- and 24-months post-randomisation for those patients enrolled in the RCT.

#### Patient experience

Patient experience is being measured by a single question adapted from the Care Quality Commission (CQC)-approved Friends and Family Test “Overall, up to this point in time how was your experience of cancer follow-up in the PETNECK2 trial?” accompanied by a free-text question “‘Please can you tell us why you gave your answer?” to capture specific details. These will be given to patients within the RCT at baseline (during randomisation, 11–14 months post-treatment) and at approximately 6-, 12-, and 24-months (if applicable) post-randomisation.

### QuinteT recruitment intervention (QRI)

A nested QuinteT Recruitment Intervention is undertaken during the internal pilot stage to qualitatively assess recruitment, identify any barriers and institute corrective measures if necessary. Changes to improve levels of informed consent and randomisation will be suggested [48]. The QRI will proceed in two iterative stages: a detailed understanding of the recruitment process will be developed in stage I, leading to tailored interventions to improve recruitment in stage II [49].

### Trial outcomes

#### Feasibility study

Five measures of feasibility were integrated into the initial part of PETNECK2, each with a defined stop/go progression criterion:

- Site recruitment: ≥4 centres to participate and achieve successful set-up (including a process for getting rapid appointments for the head and neck follow-up clinic).
- Patient recruitment: >20 eligible patients enrolled within the first six months.
- Consent rate: >20% patients providing informed consent of eligible patients approached.
- Drop-out rate: <20% of eligible patients withdrawing after completion of baseline questionnaires at any point until feasibility completion.
- Completion of baseline EQ-5D-5L and FCR Patient Reported Outcome Measures (PROMs): *≥*70% of recruited patients answering all items in the EQ-5D-5L and FCR questionnaires.

#### Randomised controlled trial

The primary outcome measure is overall survival time defined as the interval between the date of randomisation and the date of death from any cause. Patients who have not died at the time of analysis will be censored at the date when they were last known to be alive. Follow up for survival will be collected on trial Case Report Forms (CRFs), by linking with NHS Digital and other health registries and annual hospital record checks.

Secondary outcome measures include:

- Cost-effectiveness as measured by incremental cost per quality-adjusted life year (QALY) gained (combining EQ-5D-5L utility scores with overall survival data), and resource use data collection, including number and reasons for healthcare visits.
- Recurrence and distant metastasis encompassed by:
  - Disease free survival time defined as the interval between the date of randomisation and the date of a contributing event (either a recurrence (to include local, regional, and distant) or death from any cause and excluding any new primary cancer). Patients who are alive and disease-free at the time of analysis will be censored at the date when they were last known to be alive and disease-free.
  - Time from treatment to first detection of recurrence defined as the interval between the completion of the patient’s definitive preliminary treatment (not including any neck dissection that might have been implemented at three months) and a recurrence of any nature (local, regional, or distant). Patients who die before a recurrence will be censored at their date of death.
  - Details of recurrences, including location (specific details on site and whether local, regional, or distant), TNM stage at detection of recurrence and subsequent treatment for recurrence.
- Quality of life assessed by the EORTC-QLQ-C30, EORTC QLQ-H&N43, and EQ-5D-5L questionnaires.
- Fear of cancer recurrence assessed quantitively by the FCR questionnaire and qualitatively by patient interviews.
- Patient experience measured by the CQC patient experience question.
- Patient self-efficacy and motivation will be assessed using responses from the individual self-efficacy and motivation questions.
- Time to receiving an urgent review appointment, as well as the number and timings of appointments requested (for patients in the intervention arm only).

#### Randomised controlled trial’s internal pilot

An internal pilot is undertaken during the first 12-months of recruitment into the RCT. Within this, the following go/no go targets are monitored:

- Recruitment: >100 subjects over the first 12 months;
- Drop-out rate, cross over and withdrawal rates < 15%; and.
- Completion of baseline patient reported outcomes > 75%.

### Statistical analysis plan

The median overall survival time and one year (post study entry, i.e., two-year follow-up) overall survival rate for each arm will be reported. These will be calculated using the Kaplan-Meier method of estimation and presented with confidence intervals. A stratified (for stratification factors and any other key prognostic indicators e.g., smoking status) Cox proportional hazards model will be used to determine the hazard ratio and associated confidence interval, which will be evaluated against the non-inferiority margin. Analysis populations will include intention to treat (ITT), modified intention to treat (mITT) and per protocol (PP) to confirm if conclusions are consistent across the analyses. The PP analysis population will be defined as the primary analysis in this case as it is a non-inferiority study. Numbers of patients who drop out or cross over from the intervention to control will be evaluated and used to determine their inclusion in the analysis populations. Analyses that account for treatment switching will be considered if patients in the intervention arm have comparable follow-up profiles to the control. If non-proportional hazards are observed, then models accounting for non-proportional hazards will be considered and explored.

Disease free survival and time to recurrence will be presented using Kaplan-Meier plots and analysed using a stratified Cox proportional hazards model in similar fashion to the analyses proposed for overall survival. If non-proportional hazards are observed, then relevant methods will be undertaken to evaluate the intervention effect (for example flexible parametric modelling).

Recurrence details (TNM staging, location, further treatment) will be analysed descriptively and tabulated by intervention arm. Further treatments (i.e., curative intent, palliative intent, and best supportive care) will be compared between the two arms.

Qualitative data from both arms of RCT will be analysed at two time points using thematic analysis [50]. This analytical method was selected as it is a flexible approach and considers both inductive and deductive approaches employing a Leventhal’s self-regulation model to guide us as a theoretical lens [51]. The self-regulation model will help understand the patients’ illness perceptions/beliefs, symptom appraisal, and how they interact with emotional experiences such as fears about cancer recurrence. We will employ this model to guide our analyses inductively. The thematic analyses will be conducted in six phases: phases one and two: familiarisation and coding; phase three: theme development; phases four and five: reviewing and refining themes; phase six: producing the report. We will aim to explore if there are any qualitative differences in the way that the intervention helps or hinders with fears of cancer recurrence and how these fears change or do not change over time.

The overall FCR score will be analysed as a continuous outcome with summary data presented to summarise scores at the baseline and 12-month timepoints. A t-test of the change from baseline to 12 months will be assessed to determine any difference between the two arms. Linear regression will also be employed to model FCR score and adjust for any important factors.

The symptom and function scores will be calculated for each of the EORTC questionnaires returned and presented graphically and compared across the assessment time points. The overall global score from the EORTC questionnaires and the EQ-5D-5L score (and thermometer) will be compared between arms and analysed using longitudinal methods with consideration being given to missing data. Differences in all measures (overall, function and symptom) will be evaluated over time using appropriate regression analysis adjusting for baseline domain scores and other key factors where applicable. EQ-5D-5L and survival will be combined to determine QALYs for the health economic analysis.

For the patients on the intervention arm, the individual self-efficacy and motivation questions will be summarised at the evaluated timepoints and also used to determine composite scores for: (i) self-efficacy and confidence; (ii) personal motivation; and (iii) external motivation factors. These will also be correlated with intervention resource usage.

For the requested appointments descriptive reporting on the data will be performed. The duration it takes patients on the intervention arm to have a visit once it has been requested will be summarised.

The frequency and timing of the appointments requested by patients on the intervention arm will be summarised. A summary of the reasons for requesting those appointments will be provided. A comparison between intervention arms for counts of follow-up visits will be undertaken using Poisson regression.

There are no formal interim analyses.

#### Sample size determination

The sample size for the RCT has been determined assuming a baseline control overall survival proportion of 94% at 12 months (derived from H&N5000 cohort data [52] and accounting for drop out through recurrence and death during the first year after treatment prior to entering the study). The non-inferiority margin proposed is a 5% difference in overall survival, which corresponds to a hazard ratio of 1.88. Using Jungs calculation [53] and the above design parameters, in order to detect a non-inferiority margin of 5% (i.e., lower confidence interval of intervention proportion must remain above 89%) with a one-sided alpha of 2.5% and a power of 85%, 90 events would be required, and 662 patients recruited. Inflating this number to account for 5% dropout (as observed in the completed PET-NECK [33] and De-ESCALaTE [54] trials in a comparable population) a trial sample size of 698 patients is required.

### Tissue and blood samples

Although not mandated as part of the main trial, patients in the RCT component of PETNECK2 are offered the opportunity to participate in a bioresource collection sub-study. Tissue (Formalin-fixed paraffin-embedded) blocks from diagnostic biopsies and tissue from any head and neck cancer surgery, and at recurrence (if applicable), as well as blood and oral fluid samples will be collected. A 40 ml blood (plasma) sample and 10 ml oral rinse sample is taken at randomisation, 12-months post-randomisation, and at the time of recurrence (if applicable). Samples used for translational research may be used in other studies that have received ethical approval and may be transferred to other countries for testing.

All samples are collected in accordance with national regulations and requirements including standard operating procedures for logistics and infrastructure. Samples are taken in appropriately licensed premises and transported in accordance with the Human Tissue Authority guidelines and NHS trust policies.

### Adverse events reporting and analysis

There is no reason to believe that an excess of adverse events (AEs) will occur during any of the stages of PETNECK2. The collection and reporting of AEs as measured by National Cancer Institute (NCI) Common Terminology Criteria for Adverse Events (CTCAE), version 5.0 [55], is in accordance with the Research Governance Framework for Health and Social Care and the requirements of the National Research Ethics Service. Definitions of different types of AEs are listed in online Additional File 5. The reporting period for AEs is from the date of commencement of protocol defined intervention until 30 days after the completion of the interventional period.

Investigators should report AEs that meet the definition of a Serious Adverse Event (SAEs) and are deemed to be related to the study/trial intervention. The following events should not be reported as SAEs:

- Hospitalisations for:
  - Pre-planned elective procedures unless the condition worsens.
  - Treatment for progression of the patient’s cancer.
- Progression or death as a result of the patient’s cancer, as this information is captured elsewhere on the Case Report Form.

### Data management

Case Report Forms (CRF) are entered into a secure online database. Authorised staff at sites require an individual secure login username and password to access this online data entry system. For the purposes of this trial the QoL questionnaires are captured on paper and entered onto the eRDC system by the PETNECK2 Trial Office. Data reported on each CRF should be consistent with the source data or the discrepancies should be explained. If information is not known, this must be clearly indicated on the CRF. All missing and ambiguous data is queried. All sections are to be completed.

All trial records must be archived and securely retained for at least 10 years. No documents will be destroyed without prior approval from the Sponsor, via the central PETNECK2 Trial Office. On-site monitoring will be carried out as required following a risk assessment and as documented in the Quality Management Plan. In certain circumstances remote monitoring visits may be performed as an alternative to on-site monitoring visits. Additional monitoring visits may be triggered, for example by poor CRF return, poor data quality, excessive number of participant withdrawals or deviations. This may be at the request of the Trial Management Group or the Directors of the CRCTU.

Any monitoring activities will be reported to the central PETNECK2 Trial Office, and any issues noted will be followed up to resolution. PETNECK2 will also be centrally monitored, which may trigger additional on-site monitoring.

The CRCTU will hold the final trial dataset and will be responsible for the controlled sharing of anonymised clinical trial data with the wider research community to maximise potential patient benefit while protecting the privacy and confidentiality of trial participants. Data anonymised in compliance with the Information Commissioners Office requirements, using a procedure based on guidelines from the Medical Research Council (MRC) Methodology Hubs, will be available for sharing with researchers outside of the trials team within 12 months of the primary publication.

### Trial organisation structure

The University of Birmingham will act as single Sponsor for this multi-centre study: Research Governance Team, Birmingham Research Park, Vincent Drive, Edgbaston, Birmingham B15 2SQ. Email: researchgovernance@contacts.bham.ac.uk. The trial is being conducted under the auspices of the CRCTU, University of Birmingham, according to their local procedures. The TMG is responsible for clinical set-up, promotion, on-going management of the trial, interpretation of the results and preparation and presentation of relevant publications. Members of the TMG include the two Chief Investigators, the lead and trial statistician, trial management team leader and trial coordinator, members of the PETNECK2 research team responsible for delivering any part of the trial, and the PIs of the recruiting NHS sites. The TMG have quarterly meetings during recruitment.

Notwithstanding the legal obligations of the Sponsor and Chief Investigators, the Programme Management Group (PMG) is responsible for the day-to-day oversight and management of the PETNECK2 programme, including both the feasibility study and RCT. The PMG meets as required.

The Programme Steering Committee (PSC) has been established and is responsible for supervising the overall programme on behalf of the funder and the Sponsor. The PSC includes an independent chair, three independent members (one of whom represents the interest of patients and the public), and three members of the research team including the Chief Investigators. The PSC supervises the conduct of the trial, monitoring progress including pre-agreed milestones, measures of patient safety, recruitment, data completeness, losses to follow-up, and deviations from the protocol. They make recommendations about conduct and continuation of the trial to the Sponsor.

It meets at least annually with a minimum of two of the four independent members present at each meeting. The PSC provides advice through its Chair to the funder, the Sponsor, and the Chief Investigators. The PSC also act as the Trial Steering Committee (TSC).

Data analyses is supplied in confidence to an independent Data Monitoring Committee (DMC), which is asked to give advice on whether the accumulated data from the trial, together with the results from other relevant research, justifies the continuing recruitment of further patients. The DMC operates in accordance with a trial specific charter based upon the template created by the Damocles Group.

During the recruitment phase of the RCT the DMC is scheduled to meet at least annually until the trial closes to recruitment. The DMC may, at their discretion, request to meet more frequently or continue to meet following completion of recruitment. An emergency meeting may also be convened if a safety issue is identified. The DMC reports directly to the PSC, the findings of the DMC are conveyed to the funder and Sponsor. The DMC may consider recommending the discontinuation of the trial if the recruitment rate or data quality are unacceptable or if any issues are identified which may compromise patient safety.

### Confidentiality

Confidential information collected during the trial will be stored in accordance with the General Data Protection Regulation (GDPR) 2018. As specified in the PIS and with the patients’ consent, patients will be identified using only their date of birth and unique registration (feasibility study) or trial (RCT) ID number. Authorised staff may have access to the records for quality assurance and audit purposes. The Trials Office maintains the confidentiality of all patients’ data and will not disclose information by which patients may be identified to any third party other than those directly involved in the treatment of the patient and organisations for which the patient has given explicit consent for data transfer (e.g., laboratory staff).

### Dissemination of results and publication policy

A meeting will be held after the end of the study to allow discussion of the main results among the collaborators prior to publication. Results of the primary and secondary endpoints will be submitted for publication in peer-reviewed journals. Manuscripts will be prepared by the PMG, and authorship determined by mutual agreement.

### Trial status

Recruitment for the feasibility study opened in February 2022 and closed in August 2022. The feasibility study completed on 03-Apr-2023, when all feasibility patients reverted to standard of care follow-up. Recruitment for the RCT opened in January 2023.

## Discussion

Any trial proposing a substantial change to the follow-up care of HNC patients faces the potential challenge of patient and clinician acceptability. Clinicians understand that this group has significant survivorship challenges with a clear unmet need [56]. As part of the programme of work, before initiation of the RCT, we have explored these concerns with both patient and clinician workshops and individual interviews. This mixed-methods approach demonstrated support for the evaluation of a patient-led follow-up strategy. Barriers to help-seeking were identified as a potential area of risk and the RCT has been designed to try and minimise this aspect. An emphasis on patient engagement, psychosocial issues, symptom reporting, and reliability, and quick routes back to clinic were also identified as being important [37, 57]. During the feasibility trial, patients underwent qualitative interviews in an attempt to further explore their experience and potential barriers to recruitment and initiating follow-up. The results of these interviews further informed the RCT and will be published separately.

### Electronic supplementary material

Below is the link to the electronic supplementary material.

Supplementary Material 1

Supplementary Material 2

Supplementary Material 3

Supplementary Material 4

Supplementary Material 5

## Data Availability

No datasets were generated or analysed during the current study.

## Declarations

All patients provide written informed consent to participate.

## Abbreviations

AE: Adverse event
AHP: Allied health professional
CQC: Care Quality Commission
CRCTU: Cancer Research UK Clinical Trials Unit
CRF: Case report form
CTCAE: Common Terminology Criteria for Adverse Events
DMC: Data monitoring committee
FCR: Fear of cancer recurrence
HNC: Head and neck cancer
HPV: Human papillomavirus
I&S: Information and support
ITT: Intention to treat
mITT: Modified intention to treat
MRC: Medical Research Council
NCI: National Cancer Institute
NHS: National health service
OPC: Oropharyngeal squamous cell carcinoma
PET-CT: Positron emission tomography-computed tomography
PIS: Patient information sheet
PMG: Programme management group
PP: Per Protocol
PROM: Patient-reported outcome measures
PSC: Programme steering committee
QALY: Quality-adjusted life year
QRI: QuinteT recruitment intervention
RCT: Randomised controlled trial
SAE: Serious adverse event
SPIRIT: Standard Protocol Items: Recommendations for Intervention Trials
TMG: Trial management group
TSC: Trial steering committee
WHO: World Health Organization
