# Correction: PET-CT-guided, symptom-based, patient-initiated surveillance versus clinical follow-up in head neck cancer patients (PETNECK2): study protocol for a multicentre feasibility study and non-inferiority, randomised, phase III trial

**DOI:** 10.1186/s12885-024-12639-2

**Published:** 2024-07-18

**Authors:** Paul Nankivell, Piers Gaunt, Claire Gaunt, Julia Sissons, Evaggelia Liaskou, Yolande Jefferson, Tessa Fulton-Lieuw, Saloni Mittal, Hisham Mehanna, PETNECK2 Research Team, Ahmad Abou-Foul, Andreas Karwath, Ava Lorenc, Barry Main, Colin Greaves, David Moore, Denis Secher, Eila Watson, Georgios Gkoutos, Gozde Ozakinci, Jane Wolstenholme, Janine Dretzke, Jo Brett, Joan Duda, Lauren Matheson, Marcus Jepson, Mary Wells, Melanie Calvert, Pat Rhodes, Philip Kiely, Steve Thomas, Stuart Winter, Wai-lup Wong

**Affiliations:** 1 Institute of Head and Neck Studies and Education (InHANSE), Institute of Cancer and Genomic Sciences, University of Birmingham, Birmingham, B15 2TT UK; 2 Cancer Research UK Clinical Trials Unit (CRCTU), University of Birmingham, Birmingham Edgbaston, Birmingham, B15 2TT UK

**Correction** **:** **BMC Cancer 24, 823 (2024)**

**https://doi.org/10.1186/s12885-024-12470-9**

Following publication of the original article [1], it was reported that the PETNECK2 Research Team was erroneously omitted from the author group. The full list of the PETNECK2 Research Team members can be found in the Acknowledgements section.

The original article [1] has been corrected.

**Acknowledgements**

We would like to thank the PETNECK2 Patient Advisory Group for their invaluable input into the trial development and delivery, and Dr Siân Lax for contributions to the paper. Additional members of the PETNECK2 research team: Ahmad Abou-Foul (Institute of Cancer and Genomic Sciences, University of Birmingham); Andreas Karwath (Institute of Cancer and Genomic Sciences, University of Birmingham); Ava Lorenc (QuinteT research group, Bristol Medical School, University of Bristol); Barry Main (University Hospitals Bristol and Weston NHS Trust); Colin Greaves (School of Sport, Exercise and Rehabilitation Sciences, University of Birmingham); David Moore (Institute of Applied Health Research, University of Birmingham); Denis Secher (Patient Advisory Group lead); Eila Watson (Oxford School of Nursing and Midwifery, Oxford Brookes University); Georgios Gkoutos (Institute of Cancer and Genomic Sciences, University of Birmingham); Gozde Ozakinci (Health Psychology Research Group, University of Stirling); Jane Wolstenholme (Nuffield Department of Population Health, University of Oxford); Janine Dretzke (Institute of Applied Health Research, University of Birmingham); Jo Brett (Department of Midwifery, Community and Public Health, Oxford Brookes University); Joan Duda (School of Sport, Exercise and Rehabilitation Sciences, University of Birmingham); Lauren Matheson (Oxford Institute of Nursing, Midwifery and Allied Health Research, Oxford Brookes University); Marcus Jepson (QuinteT research group, Bristol Medical School, University of Bristol); Mary Wells (Nursing Directorate, Imperial College Healthcare NHS Trust, Charing Cross Hospital); Melanie Calvert (Institute of Applied Health Research, University of Birmingham); Pat Rhodes (Patient Representative); Philip Kiely (University Hospitals Bristol and Weston NHS Foundation Trust); Steve Thomas (Bristol Dental School, University of Bristol); Stuart Winter (Nuffield Department of Surgical Sciences, University of Oxford); Wai-lup Wong (East and North Hertfordshire NHS Trust, Mount Vernon Cancer Centre).
