# Supplementary material for: PET-CT-guided, symptom-based, patient-initiated surveillance versus clinical follow-up in head neck cancer patients (PETNECK2): study protocol for a multicentre feasibility study and non-inferiority, randomised, phase III trial
Source: BMC Cancer. 2024 Jul 10;24:823. doi: 10.1186/s12885-024-12470-9 (PMC11234619; doi:10.1186/s12885-024-12470-9)
Supplement: Supplementary file 2 — Supplementary Material 2 [file 12885_2024_12470_MOESM2_ESM.docx]

# Additional file 2: WHO trial registration data set for the PETNECK2 trial

| **Data category** | **Information** |
| --- | --- |
| Primary registry and trial identifying number | ISRCTN: 13709798 |
| Date of registration in primary registry | 15-Oct-2021 |
| Secondary identifying numbers | n/a |
| Source(s) of monetary or material support | National Institute for Health Research Programme Grants for Applied Research, NIHR PGfAR (reference NIHR200861) |
| Primary sponsor | University of Birmingham |
| Secondary sponsor(s) | n/a |
| Contact for public queries | PETNECK2@trials.bham.ac.uk |
| Contact for scientific queries | Paul Nankivell ([p.c.nankivell@bham.ac.uk](mailto:p.c.nankivell@bham.ac.uk))  Hisham Mehanna ([h.mehanna@bham.ac.uk](mailto:h.mehanna@bham.ac.uk)) |
| Public title | PET-CT-guided, symptom-based, patient-initiated follow-up versus regular scheduled follow-up in head neck cancer (PETNECK2) |
| Scientific title | PET-CT-guided, symptom-based, patient-initiated surveillance versus clinical follow-up in head neck cancer (PETNECK2) |
| Countries of recruitment | UK |
| Health condition(s) or problem(s) studied | Oral, oropharyngeal, nasopharyngeal, laryngeal or hypopharyngeal squamous cell carcinoma |
| Intervention(s) | Integrated feasibility study and experimental arm of randomised controlled trial: A PET-CT scan at around 12 months following completion of treatment with clinical, radiological, and pathological staging performed according to the UICC TNM Classification of Malignant Tumours staging manual 8^th^ edition. In patients with no sign of recurrence or metastases (PET-CT negative), patients will receive a face-to-face education session along with an information and support resource to monitor symptoms and treatments and will then be in control of initiating an urgent appointment when required.  Randomised controlled trial control: Standard of care clinical follow-up as per UK head and neck guidelines. |
| Key inclusion and exclusion criteria: | Ages eligible for study: Aged 18 years and over Sexes eligible for study: Both Accepts healthy volunteers: No |
|  | Inclusion criteria: Histological or cytological confirmation of oral, oropharyngeal, nasopharyngeal, laryngeal or hypopharyngeal squamous cell carcinoma; 11- to 14-months post completion of curative intent treatment by any modality (surgery, radiation, or combination treatments); no clinical symptoms or signs of loco-regional or distant metastasis, able to provide written informed consent |
|  | Exclusion criteria: Non-squamous cell carcinoma tumours, or those from sites other than those stated above, pregnant patients, clinical symptoms, or signs of loco-regional or distant metastasis, or already enrolled in a head and neck clinical trial where scheduled follow-up is required |
| Study type | Interventional |
|  | Allocation: Single arm feasibility trial following by two-arm, randomised, open-label trial |
|  | Primary purpose: Efficacy |
|  | Phase II |
| Date of first enrolment | Integrated feasibility study: 23-May-2022  Randomised controlled trial: 07-Feb-2023 |
| Target sample size | Integrated feasibility: 30  RCT: 698 (randomised 1:1) |
| Recruitment status | Integrated feasibility study: Closed to recruitment and completed (03-Apr-2023)  Randomised control trial: Open |
| Primary outcome(s) | Integrated feasibility study: Composite stop/go progression criteria of:   - Site recruitment: ≥4 centres to participate and achieve successful set-up (including a process for getting rapid appointments for the head and neck follow-up clinic). - Patient recruitment: >20 eligible patients enrolled within the first six months. - Consent rate: >20% patients providing informed consent of eligible patients approached. - Drop-out rate: <20% of eligible patients withdrawing after completion of baseline questionnaires at any point until feasibility completion. - Completion of baseline EQ-5D-5L and fear of cancer recurrence (FCR) questionnaires: >70% of recruited patients answering all items in the EQ-5D-5L and FCR questionnaires.   Randomised control trial: Overall survival time defined as the interval between the date of randomisation and the date of death from any cause. Patients who have not died at the time of analysis will be censored at the date when they were last known to be alive. |
| Key secondary outcome(s) | Randomised control trial:  • Cost-effectiveness as measured by incremental cost per quality-adjusted life year gained (combining EQ-5D-5L utility scores with overall survival data), and resource use data collection, including number and reasons for healthcare visits.  • Disease free survival time defined as the interval between the date of randomisation and the date of a contributing event (either a recurrence (to include local, regional, and distant) or death from any cause and excluding any new primary cancer).  • Time from treatment to first detection of recurrence defined as the interval between the completion of the patient’s definitive preliminary treatment (not including any neck dissection that might have been implemented at three months) and a recurrence of any nature (local, regional, or distant).  • Quality of life assessed by the EORTC-QLQ-C30, EORTC QLQ-H&N43, and EQ-5D-5L questionnaires  • Patient experience measured by the fear of cancer recurrence patient report outcomes |
