# Supplementary material for: PET-CT-guided, symptom-based, patient-initiated surveillance versus clinical follow-up in head neck cancer patients (PETNECK2): study protocol for a multicentre feasibility study and non-inferiority, randomised, phase III trial
Source: BMC Cancer. 2024 Jul 10;24:823. doi: 10.1186/s12885-024-12470-9 (PMC11234619; doi:10.1186/s12885-024-12470-9)
Supplement: Supplementary file 3 — Supplementary Material 3 [file 12885_2024_12470_MOESM3_ESM.pdf]

Additional file 3: PETNECK2 informed consent forms

To be printed on hospital headed paper

PETNECK2

Informed Consent Form – Feasibility Study

A study of PET-CT guided, symptom-based, patient-initiated surveillance versus clinical follow-up in head neck cancer

ISRCTN Number: ISRCTN13709798
IRAS Project ID Number: 298368
Sponsor Protocol Number: RG_21-035

Please initial each box to confirm participation in the research:

1. I confirm that I have read and understand the Patient Information Sheet/s (Version ___, dated ______________) for the PETNECK 2 feasibility study. I have had the opportunity to consider the information, ask questions, and have had these questions answered satisfactorily. ☐

2. I understand that my participation is voluntary and that I am free to withdraw at any time without giving a reason, without my medical care or legal rights being affected. ☐

3. I give permission for my personal details (e.g. name, patient initials, date of birth, hospital number, address and post code, phone number and email address, racial/ethnic origin) to be given to the PETNECK 2 Trial Office and Research Team as well as a copy of this consent form. ☐

4. I understand that information collected for this study is covered by the EU General Data Protection Regulation (GDPR) and Data Protection Act (2018) and all information will be stored securely. ☐

5. I understand that relevant sections of my medical notes and data collected during this study may be looked at by individuals within the PETNECK 2 Trial Office and Research Team, the Sponsor (University of Birmingham), the funder, regulatory authorities and/or the lead NHS Trust, where it is relevant to my taking part in this research. These records may need to be accessed remotely. I give permission for these individuals to have access to this data. ☐

Continue on next page

Informed Consent Form – Feasibility Study

Please <u>initial</u> each box to confirm participation in the research:

6. I agree for data relating to my involvement in the study identified above, and other relevant data collected, to be transferred between the Universities of Birmingham, Bristol, Stirling and Oxford Brookes, where members of the PETNECK 2 Research Team are based, and for the data to be stored securely for 10 years at the University of Birmingham where only immediate members of the PETNECK 2 Research Team will have access to it. ☐
7. I agree for my App/website and booklet usage and health data in the Information resource to be collected by the PETNECK 2 Research team for purposes of conducting research. ☐
8. I agree to provide my email address to Endoscope-i when activating my App/website account for the purposes of being able to send a confirmation of sign-up and reset a forgotten password. ☐
9. I understand that anonymised data from my participation in this study, including the use of appropriate quotes, may be used in publication(s) of the research findings. ☐
10. I agree to my GP being informed about my participation in the study ☐
11. I agree to the use of audio-recordings in the interview. I understand that my name will not be attached to the recording. ☐
12. I agree to take part in this study. ☐

Original to be kept in the Investigator Site File, 1 copy in hospital notes, 1 copy to the patient, 1 copy to the Trial Office

Informed Consent Form – Feasibility Study

The following are optional and will not affect entry into the study. Please enter your initials in the relevant boxes if you agree:

13. I give permission for a copy of my PET-CT scan, (if applicable) labelled with my study number only, to be shared with the PETNECK 2 Research Team. I understand that this scan may be used for future ethically and scientifically approved research studies. I understand that my pseudoanonymised clinical data may be supplied with the scans. ☐

14. I agree to Oxford Brookes contacting me to discuss participating in a PhD research study involving an informal interview about how patients feel about checking for symptoms after head and neck cancer. ☐

**Name of patient**	**Signature**	**Date**
**Name of person taking consent**<br>You must have signed the<br>Site Signature & Delegation Log	**Signature**	**Date**

CRCTU-ICF-QCD-001, version 2.0a

Original to be kept in the Investigator Site File, 1 copy in hospital notes, 1 copy to the patient, 1 copy to the Trial Office

IRAS Project ID Number: 298368
Version 3.0, 26th July 2022

To be printed on hospital headed paper

PETNECK2

Informed Consent Form – Randomised Controlled Trial

Trial of PET-CT guided, symptom-based, patient-initiated surveillance versus clinical follow-up in head neck cancer

ISRCTN13709798
IRAS Project ID Number: 298368
Sponsor Protocol Number: RG_21-035

Site: ....

Patient's TNO: | | | | |

Principal Investigator: ....

Please initial each box

1. I confirm that I have read and understand the Patient Information Sheet **(version .... dated...........................)** for the above study. I have had the opportunity to consider the information, ask questions and have had these answered satisfactorily. ☐

2. I understand that my participation is voluntary and that I am free to withdraw at any time without giving any reason, without my medical care or legal rights being affected. ☐

3. I give permission for my name, initials, date of birth, race/ethnic origin, contact details including phone number, email address, and postal address, hospital number and NHS number (or in Scotland the Community Health Index (CHI) or in Northern Ireland the Health & Care (H&C)) number to be given to the PETNECK2 Trial Office and Research Team when I am entered into the study, as well as a copy of this Consent Form. ☐

4. I understand that information collected for this study is covered by EU General Data Protection Regulation (GDPR) and Data Protection Act (2018) and information will be stored securely. ☐

5. I understand that relevant sections of my medical notes and data collected during the study may be looked at by individuals from the PETNECK2 Trial Office and Research Team, regulatory authorities, the Sponsor (University of Birmingham) and/or NHS bodies, where it is relevant to my taking part in this research. These records may be accessed remotely. I give permission for these individuals to have access to my records. ☐

Original in the Investigator Site File, 1 copy in hospital notes, 1 copy to the patient, 1 copy to Trials Office, 1 copy to GP

CONFIDENTIAL ON COMPLETION

Informed Consent Form – Randomised Controlled Trial

Please initial each box

6. I understand that the PETNECK2 Trial Office may access information held by Cancer Registries and/or the national health registries to keep in touch with me and to follow up on my health status. ☐

7. I agree for data relating to my involvement in the study identified above, and other relevant data collected, to be transferred between the Universities of Birmingham, Bristol, Stirling and Oxford, where members of the PETNECK2 Research Team are based, and for the data to be stored securely for 10 years at the University of Birmingham where only immediate members of the PETNECK2 Research Team will have access to it. ☐

8. I agree to provide my email address to Endoscope-i when activating my App/website account for the purposes of being able to send a confirmation of sign-up and reset a forgotten password. ☐

9. I agree for my App/website and booklet usage in the Information resource to be collected by the PETNECK2 Research team for purposes of conducting research. ☐

10. I agree to my GP being informed of my participation in this study. ☐

11. I agree to take part in the PETNECK2 study. ☐

Original in the Investigator Site File, 1 copy in hospital notes, 1 copy to the patient, 1 copy to Trials Office, 1 copy to GP

CONFIDENTIAL ON COMPLETION

The following are OPTIONAL and will not affect entry into the study. Please enter your initials in the relevant box if you agree:

Please initial the selected option

	No	Yes
12. I give permission for a copy of all scans (CT, MRI, PET-CT and other scans) (if applicable) only labelled with my trial number, to be shared with the PETNECK2 Research Team. I understand that this scan may be used for future ethically and scientifically approved research studies. I understand that my pseudoanonymised clinical data may be supplied with the scans.		

Options 13-15 relate to the PETNECK2 study.

	No	Yes
13. I agree to provide samples of blood for research associated with this study and understand that any blood samples that I donate will be labelled with my trial number, and will be sent to the University of Birmingham, for storage for research associated with this trial.*		
14. I agree to provide samples of oral fluid for research associated with this study and understand that any oral fluid samples that I donate will be labelled with my trial number, and will be sent to the University of Birmingham, for storage for research associated with this trial.*		
15. I agree to the collection of tissue from both my diagnostic biopsy and remaining samples from any future surgery or future biopsy being used for research purposes and understand that any tissue samples that I donate will be labelled with my trial number, and will be sent to the University of Birmingham, for storage for research associated with this trial.*		

**I understand that giving tissue samples is a gift for this research, is voluntary and that I am free to withdraw my approval for use of the samples at any time without giving a reason and without my medical treatment or legal rights being affected by this voluntary donation.* (*)

Options 16-18 relate to possible future research.

	No	Yes
16. I agree to be contacted by the Research Team to discuss my participation in future related studies.		
17. I understand that research may include genetic, current and future tests and analyses of my samples aimed at understanding the factors that may cause and influence my type of cancer and its treatment, as well as helping in selecting treatment response. I understand that I will not be informed of the results of these studies.		
18. I agree that my data, my samples and scans which remain at the end of this study can be stored for use in future ethically and scientifically approved research in the UK or internationally, including genetic studies, artificial intelligence studies for healthcare purposes, research that may use animals or in vitro models, and research involving academic, private or		

commercial companies. I understand that my pseudoanonymised clinical data may be supplied with the samples.

Name of patient	Signature	Date
Name of person taking consent<br>You must have signed the Site Signature & Delegation Log	Signature	Date
