# Supplementary material for: PET-CT-guided, symptom-based, patient-initiated surveillance versus clinical follow-up in head neck cancer patients (PETNECK2): study protocol for a multicentre feasibility study and non-inferiority, randomised, phase III trial
Source: BMC Cancer. 2024 Jul 10;24:823. doi: 10.1186/s12885-024-12470-9 (PMC11234619; doi:10.1186/s12885-024-12470-9)
Supplement: Supplementary file 4 — Supplementary Material 4 [file 12885_2024_12470_MOESM4_ESM.pdf]

Additional file 4: PETNECK2 patient information sheets

To be printed on hospital headed paper

Patient Information Sheet - Feasibility Study

PETNECK2

ISRCTN Number: ISRCTN13709798

IRAS Project ID Number: 298368

Sponsor Protocol Number: RG_21-035

Based on the progress with your treatment, your clinical team would like to offer you the opportunity to be part of a clinical study called PETNECK 2.

This study can help influence the future direction of follow-up for head & neck cancer patients. It is important and we would greatly value your contribution.

Your doctor or nurse will also go through this information sheet with you and answer any questions you may have. Some of the language below may be a bit "medical". We have tried to keep it clear and simple, but please don't be afraid to ask a member of your clinical team. You may also want to discuss this information sheet with your friends and relatives.

Take your time to decide whether or not you want to take part. If you decide not to take part, this will not affect the type or quality of the care you receive.

What is the purpose of the PETNECK2 Feasibility Study?

The purpose of the feasibility study is to test a new patient-led follow-up method for low risk head and neck cancer patients in a small number of patients before testing in a larger clinical study.

Currently, people who finish treatment for head and neck cancer attend a routine clinic appointment for review every two to six months for five years to check if the cancer has come back (called *recurrence*). If caught early, there is a better chance of successful treatment. However, most patients feel they are seen too frequently, which can increase their worry about cancer recurrence. It is also inefficient because few cancers are found this way.

Studies show that cancer recurrence is much more likely to be found when patients ask to be seen because their symptoms have changed.

Studies also show that a type of scan, called PET-CT, done one year after treatment can identify patients who are unlikely to get recurrence and could therefore be followed up less frequently.

We have developed a new PET-CT guided, patient-led way to follow up patients who have a low risk of their cancer coming back (called recurrence). We propose patients have a PET-CT scan one year after finishing treatment. If no cancer is detected, patients will be educated by a nurse or AHP (Clinical Nurse Specialist, Speech and Language Therapist or Dietitian), about what symptoms of recurrent cancer to look out for. The information will also be given as a mobile App/website or paper booklet. Patients will then be given an 'open urgent appointment' guaranteeing review by their clinical team **within 2 weeks** if they develop worrying symptoms. They will not have to have regular clinic visits.

Before testing this new follow-up in a large clinical study, we would like to see how well it works in a smaller number of patients. Thirty eligible patients will take part in this study around the UK.

What is the new follow-up method being tested?

The new follow-up method includes having a PET-CT scan at around one year after treatment. If that is clear, then you would have an education session with an Allied Health Professional (AHP) (Clinical Nurse Specialist, Speech and Language Therapist or Dietitian) or Nurse who will go through and explain what to look for in terms of symptoms that suggest the cancer may have come back, and they will also give you information in written form (by App, website or paper booklet according to your preference). They will also explain how to use them and what to do in case you have any concerns and wish to contact or see your clinical team.

You will not have scheduled clinical follow-up appointments, but will be given an 'open urgent appointment' where you **will be seen** in the normal head and neck follow-up clinic **within two weeks** of contacting the clinical team, if any new or worrying symptoms appear. Therefore, you will not reduce your access to the clinical team but will, in effect, have access to them according to your need.

We will also collect your opinion on this new method of patient-led follow-up and on the information and support package provided to help you while on patient-led follow-up. This will help us to improve this new patient-led follow-up method before we test it in a larger clinical study.

As part of the feasibility study, you will be asked to test some of the questionnaires that we plan to use in the study. These questionnaires measure your quality of life, resource use and any concerns you may have on the cancer coming back. In the feasibility study the information will be used to see how you find completing the questionnaires. Your study nurse will explain how you will be able to complete the questionnaires at clinic and home.

What is a PET-CT scan?

A Positron Emission Tomography-Computed Tomography (PET-CT) scan are images taken by a relatively new type of scanner, called a PET-CT scanner, which takes pictures of the tissues and cells inside the body. Because cancer cells are much more active than other cells, the PET-CT can detect this activity. More importantly, they are good at finding hidden cancers left behind after treatment and

at detecting the absence of cancer cells in the neck. With your consent, a copy of your PET-CT scan will be held by the PETNECK2 Research Team (based at the University of Birmingham), and may be used for future research that has been approved by an ethics committee.

What is the Information Resource?

The Information Resource will be given to you during a face-to-face education session delivered by an AHP or Nurse. This resource will be an App, which can be downloaded onto your mobile phone, laptop, and/or tablet or it can also be viewed on a website. If you are not comfortable with using the App or website, you will be given a paper-based booklet. The information resource will include:

- information on key symptoms of recurrence that you should look out for
- techniques to check yourself and reminders to check your symptoms
- how to make an urgent appointment if you find new or worrying symptoms
- how to overcome any concerns you might have about being on patient-led follow up
- links to HNC peer support groups and cancer charities
- links to existing information about living well after a head and neck cancer diagnosis and managing side effects
- contact details of your head and neck cancer clinical team and other key healthcare professionals
- a diary for you to record your symptoms

What will happen to me if I take part?

This information sheet explains what the study is about. Your doctor and/or research nurse will also discuss the study with you and answer your questions. If, after having had time to consider this information and ask any questions, you decide to take part, you will be asked to sign the Informed Consent Form and will be given a copy of the signed form for your record. Once you have done this, you will be registered in the study. Your hospital will then request for you to have a PET-CT scan at around 12 months from completion of your treatment. You will also be asked to complete some questionnaires in the clinic. Your study doctor or research nurse will explain to you how to complete these questionnaires. These questionnaires measure your quality of life, resource use and any concerns you may have on the cancer coming back.

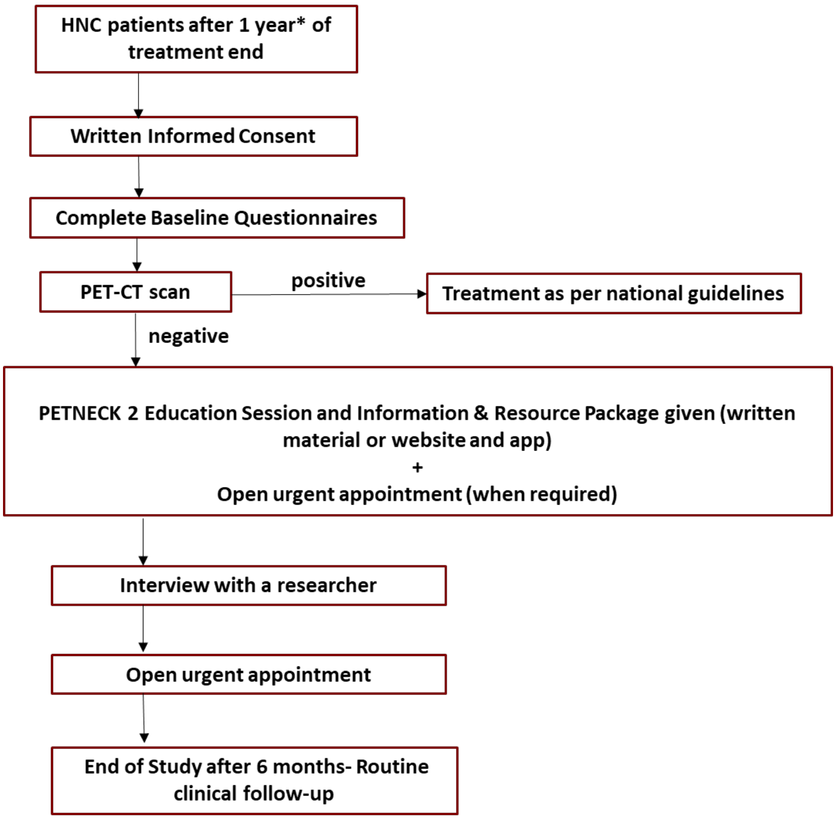

* 1 year after treatment end can apply from 11 – 14 months post-treatment

Once the PET-CT scan results are available, your study doctor or a member of your hospital team will contact you to discuss the results.

If the PET-CT scan is positive or unclear, you will undergo further tests as recommended by UK national guidelines. If further tests confirm a recurrence, your study doctor will discuss treatment options with you. You will return to regular scheduled follow-up but we will collect information about your treatment as part of the study, for the duration of the feasibility study. If the PET-CT scan is negative (no sign of recurrence or cancer spreading), or if after you have had further investigations and these rule out recurrence, you will be asked to return to the hospital for a PETNECK 2 Education session with a Clinical Nurse Specialist, Speech and Language Therapist, dietician or other Allied Health Professional. This session will last approximately 30-60 minutes. Before this education session, you will also receive a link to watch a video that tells you more about the study, shows you how to look for new and important symptoms and also gives you instructions on how to download the PETNECK 2 App on your phone/tablet or access the website. **During the PETNECK 2 Education session, the following will be covered:**

- Explore and discuss any concerns you might have about being on patient-led follow up
- Help you download the App, if needed

- Show you how to use the App and website, or review the paper booklet
- Discuss what is normal for you and symptoms of recurrence to look out for
- Show you how to check for any changes or new symptoms
- Tell you about the excellent additional support available from online and local support groups and charities, available through the App or paper booklet
- Add and check the contact details of your local head and neck cancer clinical team, including your key healthcare worker; tell you who to contact about concerns, and show you how to contact them
- Discuss how to make a review appointment at any time (called an 'open urgent appointment')

Around one to two months after the PETNECK 2 Education session:

Approximately two to three weeks after your PETNECK 2 Education session, a PETNECK 2 Researcher will get in contact with you to arrange an interview. This interview will take place approximately one to two months after your education session. You can choose to be interviewed either face-to-face, via videocall, email or over the phone, and at a time convenient to you. The interview will last approximately one hour, and there are no right or wrong answers. We are interested in learning about your experience of using the App, website and/or paper booklet and any suggestions you might have to improve them. We will also want to know what you think about this new way of follow-up as well as your opinions about the tools you tested. The interview will be audio/video recorded, and later typed up and analysed by researchers working on the study. The transcript will be anonymised at the point of being typed up, so any personal data (names, places etc.) that might identify you will be removed. Your answers will be combined with those of other patients to help us to improve the content of the Information Resource.

You will also receive study questionnaires (similar to the one you will fill out at the first study appointment) to complete either electronically or by post along with a pre-paid self-addressed envelope, which you will need to post back.

Your participation in the study will continue until the end of the feasibility study, after which you will return to regular clinical follow-up.

What are the benefits to taking part?

A possible benefit of taking part is to contribute to improving follow up care of future head and neck cancer patients.

The PET-CT scan can, if positive, detect a recurrent cancer earlier than if you did not have one or, if negative, will provide some reassurance to you that you don't have cancer at that time.

It also gives you access to the latest technology in checking the recurrence of cancer (PET-CT scan), which is not currently available as a routine clinical practice at one year after end of treatment.

Research has shown that if cancer is detected earlier there are better chances of successful treatment. You will have an OPEN URGENT APPOINTMENT so that you are able to see your clinical team **within 2 weeks** of reporting/contacting your clinical team if any new or worrying symptoms appear.

You may find patient-led follow-up more flexible and tailored to your needs as less frequent clinical visits may save you time and money in travelling to the hospital.

What are the possible disadvantages and risks to taking part?

You will have a Positron Emission Tomography-Computed Tomography (PET-CT) scan as part of your participation in study. This will be extra to any other scan that you would have if you did not take part in the study. This procedure uses ionising radiation to form images of your body and provide your doctor with other clinical information. Ionising radiation may cause cancer many years or decades after the exposure. We are all at risk of developing cancer during our lifetime. 50% of the population is likely to develop one of the many forms of cancer at some stage during our lifetime. Taking part in the feasibility study will increase the chances of this happening to you to about 0.07%.

It is possible that discussing issues surrounding your cancer and follow-up care may be challenging and may cause you distress. The interview will be conducted by an experienced and sensitive researcher and you don't have to answer anything you are not comfortable discussing. However, if you do have any distress after taking part in the interview, please contact your Clinical Nurse Specialist.

Do I have to take part?

No, it is up to you to decide whether or not to take part in this study. Your participation is entirely voluntary. If you decide to take part, you are free to withdraw at any time without having to give any reason for your decision. This will not affect the standard of care you receive.

Will my taking part in this study be kept confidential?

All information that is collected about you will be subject to the EU General Data Protection Regulation and to the Data Protection Act 2018 for health and social care research and will be kept confidential and nothing that might identify you will be revealed to any third party other than those involved in your

care or this research. Access to your personal information will be restricted to members of the PETNECK 2 Research Team and the PETNECK 2 Trial Office within the Cancer Research UK Clinical Trials Unit for the purposes of the research.

A copy of your PET-CT scans will be pseudo-anonymised by your hospital and labelled with your study number only before they are transferred by CD to the PETNECK 2 Research Team, based at the University of Birmingham, for future ethically approved research. The original scan will be retained by your hospital. In extremely rare instances, patient identifiers may be hidden into the coding of the images dependant on the supplier of the scanning equipment. The images will be retained for future research and when the images are sought for use, they will be individually checked by the local clinical team (independent to the research team), to ensure that all identifiers are removed before any further research is conducted.

Any information that you enter into the App/website or booklet with be confidential and no one will be monitoring this. However, it would be helpful for us to understand how well patients interact with the App/website or booklet and therefore we will get information on the number of times you interact with the App/website over the period of the study.

You will need to provide your email address when activating your account for the App/website. This is to ensure that endoscope-i (App developers) can send you a confirmation of sign-up and a link to reset your password in case you forget it.

The audio/video recordings of your interview and other study documents will be saved using your registration ID number and not linked to your name. We may use your words in reports, publications or presentations arising from the study, but we will never use your name or information that will allow you to be identified.

Who is the Data Controller?

The University of Birmingham (UoB), Edgbaston, Birmingham B15 2TT and Oxford Brookes University, Marston, Oxford OX3 0FL are the data controllers and/or processors for the personal data that we process in relation to you. We will be using information from you in order to undertake this study. This means that we are responsible for looking after your personal information and using it properly.

By taking part in the study, you will be agreeing to allow members of the research staff at your hospital and from the PETNECK 2 Trial Office to look at your study records, which includes your medical records. It may also be necessary to allow the authorised personnel from government regulatory agencies, the Sponsor and/or NHS bodies to have access to information about you. This is to ensure that the study is being conducted to the highest possible standards.

The legal justification we have under data protection law for processing your personal data is that it is necessary for our research, which is a task we carry out in the public interest. This data will not be used to make decisions about you. All individuals who have access to your information have a duty of confidentiality to you.

If you choose to withdraw from the study, we would still like to collect relevant information about your health. If you have any objection to this please let your study doctor know.

Under no circumstances will you be identified in any way in any report, presentation or publication arising from this study.

Your rights to access, change, or move your information are limited, as we need to manage your information in specific ways in order for the research to be reliable and accurate. If you withdraw from the study, we will keep the information about you that we have already obtained.

To safeguard your rights, we will use the minimum personally-identifiable information possible. Under the provisions of the EU General Data Protection Regulation, you have the right to know what information the PETNECK 2 Trial Office have recorded about you. If you wish to view this information, please contact Legal Services at the address below. Please note that a small fee may be payable to retrieve this information.

Legal Services

The University of Birmingham

Birmingham

B15 2TT

You can find out what we do with your data in our Privacy Notice see:

https://www.birmingham.ac.uk/crctu

Where will my data be stored?

We will need to use information from you for this research project.

This information will include your name, initials, date of birth, contact details (address and post code, email address and phone number), racial/ethnic origin and hospital name as well as data obtained as part of the study. Your name, address and contact details will be shared with and transferred between the researchers at the University of Birmingham and Oxford Brookes University. PETNECK 2 Researchers from these organisations will use this information to arrange qualitative interviews and send out research questionnaires. The University of Birmingham will also share relevant data collected about you with the researchers at Oxford Brookes University for the purpose of analysing the information collected at interview. The researcher will be provided with your age, gender and ethnic background (taken from the first questionnaire that you completed at the first study appointment). The researcher will also be provided with the type of head and neck cancer you were diagnosed with and what treatment you were given. This is so that when reviewing the information they can check whether the support resource given to you is appropriate for all type of head and neck cancer, treatments, groups, ages, genders and ethnic groups. It is important that the support resource caters for all patients diagnosed with head and neck cancer.

Researchers will use the information you have provided in the completed research questionnaires to conduct health economics analysis, this is a field that looks at understanding and promoting health for all. For this, researchers may also require socioeconomic data such as ethnicity, and smoking and alcohol consumption status, as well as key information related to your treatment pathway.

A copy of your PET-CT scans will be pseudo-anonymised by your hospital and labelled with your study number only before they are transferred by CD to the PETNECK 2 Research Team, based at the University of Birmingham, for future ethically approved research. The original scan will be retained by your hospital. In extremely rare instances, patient identifiers may be hidden into the coding of the images dependant on the supplier of the scanning equipment. The images will be retained for future research and when the images are sought for use, they will be individually checked by the local clinical team (independent to the research team), to ensure that all identifiers are removed before any further research is conducted.

People who do not need to know who you are will not be able to see your personal details or contact details. Your data will have a unique registration number instead.

The data about you (including your anonymised interview transcript) will be stored on secure university networks at the University of Birmingham and Oxford Brookes University, in accordance with the Data Protection Act 2018, and the Universities data management policies. We will keep all information about you safe and secure.

Long-term storage of the data will be at the University of Birmingham; the data will be stored for 10 years after the study has finished. If you withdraw from the study, we will keep the information we have already obtained but, to safeguard your rights, we will use the minimum personally-identifiable information possible.

Where can you find out more about how your information is used?

You can find out more about how we use your information

- at www.hra.nhs.uk/information-about-patients/
- our leaflet available from the HRA website at www.hra.nhs.uk/patientdataandresearch
- by asking one of the research team
- by sending an email to the PETNECK 2 Programme Manager at s.mittal.2@bham.ac.uk, or
- by contacting the Sponsor's Data Protection & Freedom of Information Manager at dataprotection@contacts.bham.ac.uk

What if there is a problem?

In the unlikely event of you have a concern about any aspect of this study, you should ask to speak to your research doctor or nurse who will do their best to answer your questions. You can use the contact number at the end of this sheet.

Complaints

If you have a concern about any aspect of this research study, you should contact your study doctor in the first instance. You can use the contact number at the end of this sheet.

If you remain unhappy and wish to complain formally, you can do this through the Patient Advice and Liaison Services (PALS) or the National Health Service (NHS) complaints procedure. Details can be obtained from your hospital.

Harm

In the event that something does go wrong and you are harmed and this is due to negligence then you may have grounds for legal action for compensation against the sponsor of the study (University of Birmingham) or the NHS Trust but you may have to pay your legal costs, as would be the situation even if you were not in a study. NHS Trust and Non-Trust Hospitals have a duty of care to patients treated, whether or not the patient is taking part in a study and the normal NHS complaints mechanisms will still be available to you. The Sponsor of the study does not hold insurance against claims for compensation for injury caused by participation in this study and they cannot offer any indemnity.

Will my GP be involved?

It is important that your GP is kept up to date with any research study you are participating in. With your permission your GP will be informed that you are taking part in this feasibility study.

What will happen to the results of the PETNECK 2 Feasibility Study?

The results from the PETNECK 2 study will be used to make changes to our App, website and booklet that supports and educates patient about PET-CT guided patient-led follow-up. The results of this feasibility study will also help us to refine the design of our larger clinical study to test this new follow-up method against the standard regular clinic follow-up.

Reports of the study findings and academic papers may be published. Findings may also be presented at academic conferences, workshops, or used for training purposes. You will not be identified in any report, publication, or presentation.

Who is organising and funding the research?

The research is being organised and run by the University of Birmingham in collaboration with the Universities of Bristol, Oxford, Oxford Brookes University, and Stirling. The feasibility study is being coordinated by the Cancer Research UK Clinical Trials Unit at The University of Birmingham.

The sponsor of the study is the University of Birmingham. This study is funded by the National Institute for Health Research (NIHR) under its Programme Grants for Applied Research Programme (project reference NIHR200861).

The views expressed are those of the author(s) and not necessarily those of the NIHR or the Department of Health and Social Care.

Additional Research

The Oxford Brookes University researchers, who are conducting the interviews in the feasibility study, are also conducting a PhD research study. This study will explore how confident patients and their caregivers feel about checking their symptoms after a diagnosis of head and neck cancer and their experiences of checking for symptoms. With your consent, the research team at Oxford Brookes University would like to contact you to provide information about this PhD research study, which would involve an informal interview (telephone or online) with a researcher lasting approximately 45-60 minutes. There is no obligation to take part in this PhD study.

Expenses and payments

You will not receive any money for taking part in this research study; however, we will reimburse travel and parking expenses to attend the interview, if it takes place outside of your home.

Your study doctor, research nurse or Allied Health Professional will not receive any payments for including you in this study.

Who has reviewed the study?

All research in the NHS is looked at by an independent group of people, called a Research Ethics Committee, to protect your safety, rights, wellbeing and dignity. This study has been reviewed and given favourable opinion by a Research Ethics Committee. It has also been reviewed by the Research and Development Office at your hospital.

Further Information and Contact Details

What happens now?

You will have some time to think about the study and make your decision. You may wish to discuss it with your family or friends. If you take part, you will receive a copy of this information sheet and a copy of the signed consent form to take home. It is possible that your consent might be taken before you reach your 12 months post treatment appointment. In this case please be aware that there might be a few weeks between agreeing to take part and anything happening. Your Study doctor or nurse will need to re-confirm that you still can proceed before your scan at around 12 months after you last received treatment. This may include a call by, or visit to, your clinical team or to the study team. Your Study doctor will also inform your GP of your decision to take part in the study. If, at any time, you have any questions about the study you should contact your study doctor or research nurse using the details below.

Local contact details:

Study Doctor: ____

Research Nurse: ____

☎: ____

Emergency (24 hours)

☎: ____

You may also find it helpful to contact the following organisations:

Your local Patient Advice and Liaison Service (PALS) or local equivalent who provide advice and support to patients, their families and their carers; website: http://www.nhs.uk

The contact number of your local Patient Advice and Liaison Service or local equivalent is:

CancerHelp, an information service about cancer from Cancer Research UK, Freephone 0808 800 40 40, website: www.cancerhelp.org.uk

Macmillan Cancer Support: Freephone 0808 800 0000, website: www.macmillan.org.uk

You can also contact your local hospital team:

[Sites to enter name, address, email address, telephone numbers including the 24 hour emergency contact number].

[Delete as appropriate]Should you have any complaints you can contact the Patient Advice and Liaison Services (PALS) or the National Health Service (NHS). Your local PALS is:

[Enter local details]

[Delete as appropriate] In Northern Ireland the Patient Client Council (PCC) can provide assistance and support at any stage of the health and social care services complaints procedure. The PCC is an independent body who represent the views of the public in all areas of health and social care. They can also assist you to make a complaint. This is a confidential and free service.

http://www.patientclientcouncil.hscni.net/

Telephone: 0800 917 0222

Email: info.pcc@hscni.net

[Delete as appropriate] The Patient Advice and Support Service is an independent service which provides free, accessible and confidential information, advice and support to patients, their caregivers and families about NHS healthcare in Scotland.

http://www.patientadvicescotland.org.uk/

[Delete as appropriate] Community Health Councils (CHCs) are independent bodies, set up by law, who listen to what individuals and the community have to say about the health services with regard to quality, quantity, access to and appropriateness of the services provided for them. CHCs can also help, advise and support people who wish to make complaints about NHS services and similar matters. This advice is completely free, independent and confidential.

http://www.wales.nhs.uk/sitesplus/899/page/99722

Thank you for taking the time to read this Information Sheet.

To be printed on hospital headed paper

Patient Information Sheet

Trial of PET-CT guided, symptom-based, patient-initiated surveillance versus clinical follow-up in head neck cancer

ISRCTN13709798

IRAS Project ID Number: 298368

This is your chance to make a real difference!

Based on the progress with your treatment, your clinical team would like to offer you the opportunity to be part of a clinical study called PETNECK2.

This study can help influence the future direction of follow-up for head and neck cancer patients. It is important and we would greatly value your contribution.

The study will look to see how good a new patient-led approach for follow-up is and compare it to the existing standard clinic-led schedule of appointments.

The clinic-led approach is the current approach that you are already following and with which you will already be familiar. This involves being seen every 3-6 months in the clinic for a routine follow-up appointment.

The patient-led approach will be explained in detail in this document. Essentially it involves getting a special scan (called a PET-CT scan) which helps detect any recurrences early, then the patient having more involvement with their follow-up and greater control over their appointment schedule.

Your doctor or nurse will also go through this information sheet with you and answer any questions you may have. Some of the language below may be a bit "medical". We have tried to keep it clear and simple, but please don't be afraid to ask any questions to a member of your clinical team. You may also want to discuss it with your friends and relatives.

There are two parts to this information sheet:

- Part 1 explains the purpose of the study and what will happen to you if you decide to take part
- Part 2 provides you with more detailed information about how the study is run

Take your time to decide whether or not you want to take part. If you decide not to take part, this will not affect the type or quality of the care you receive.

Part 1

You are being invited to take part in a research study called PETNECK2

Before you decide if you want to take part, it is important for you to know why this research is being done and what it would involve for you. Please take some time to read this information about the PETNECK2 study, and talk it through with others (friends, family, your GP) if you wish. If anything is unclear, you would like more information, or you have any concerns, please do not hesitate to ask a member of your clinical team. Their contact details can be found at the end of this information sheet.

What is the purpose of the PETNECK2 study?

The purpose of the PETNECK2 study is to compare two methods of follow up to try to find out whether one is better than the other. This is because we do not currently know what is the best way to follow up head and neck cancer patients.

Why is there a need for the study?

Currently, people who finish treatment attend a routine clinic appointment for review every three to six months for five years to check if the cancer has come back (called *recurrence*). If *recurrence* is caught early, there is a better chance of the patient being successfully treated. However, most patients feel they are seen too frequently, which can increase their worry about cancer recurrence. It is also inefficient because few cancers are found this way.

Studies show that cancer recurrence is much more likely to be found when patients ask to be seen because their symptoms have changed.

Studies also show that a type of scan, called PET-CT, done one year after treatment can identify patients who are unlikely to get recurrence and could therefore be followed up less frequently.

Why have I been invited to take part in PETNECK2?

You are being invited to take part because your clinical team (doctors and nurses) think that you would be suitable for the study. They have identified you as someone who may meet the entry requirements

for the study. They are happy for you to take part and contribute to this national research study that may change the way head and neck cancer patients like yourself are followed up in future.

Do I have to take part?

No, taking part in research is always optional. If you do not take part, this will not affect the type or quality of the care that you receive. If you decide to take part, you will be asked to sign a consent form to document your agreement to participate. You are free to leave the study at any point without giving a reason and, again, this will not affect your care.

What are the follow-up methods being compared?

a) PET-scan guided, patient-led follow up

We have developed a new PET-CT guided, patient-led way to follow up head and neck cancer patients who have a low risk of their cancer coming back (called recurrence). We would like to test this new patient-led follow-up method and compare it with the current method of follow-up.

The new follow-up method includes having a PET-CT scan at around one year after treatment. If that is clear, then you would have an education session with an Allied Health Professional (AHP) (Clinical Nurse Specialist, Speech and Language Therapist or Dietitian) or Nurse, who will go through and explain what to look for in terms of symptoms that suggest the cancer may have come back. They will also give you information in written form (by App, website or paper booklet according to your preference) and they will explain how to use them and what to do in case you have any concerns and wish to contact or see your clinical team.

You will not have scheduled clinical follow-up appointments, but will be given an ‘open urgent appointment’ where **you will be seen** in the normal head and neck follow-up clinic **within two weeks** of contacting the clinical team. Therefore, you will not reduce your access to the clinical team but will, in effect, have access to them according to your need.

b) Clinic-led follow up

The current follow up method involves patients attending a clinic review every three to six months for four years.

What will happen to me if I take part?

Six hundred and ninety eight (698) eligible head and neck cancer patients across the UK will take part in this study.

If, having read this information sheet and you've had your questions answered, you are willing to take part in the study, you will be asked to sign the Informed Consent Form (ICF) and will be given a copy.

Once you have signed the consent form, you will be registered into the study. This can happen between 6-12 months from the end of your head and neck treatment. When/if you are within the 11-14 months study window you will then be allocated to one of two groups:

Group A: Patient-led follow-up or

Group B: Clinic-led follow-up

To compare the two follow-up methods, half of these patients will have patient-led follow up, and half of the patients will have clinic-led follow up. To make sure this is done fairly, the method of follow-up is allocated by a process called randomisation. This means that you will be allocated to either the patient-led follow-up or clinic-led follow-up group by a computer programme based at the Cancer Research UK Clinical Trials Unit. It is important that you only agree to take part in the study if you would be prepared to accept either follow-up method, because neither you nor your doctor/nurse will be able to choose which group you go in to. Please note, that the patient-led follow-up method is not currently offered outside of research studies.

Before you are allocated to a Group you will be asked to complete some questionnaires in the clinic. Your study doctor or research nurse will explain to you how to complete these questionnaires. These questionnaires measure your quality of life, any concerns you may have on the cancer coming back, use of healthcare resources, questions about your self-checking habits, how confident you feel about carrying out self-checks and how confident you feel that your concerns will be addressed by your clinical team.

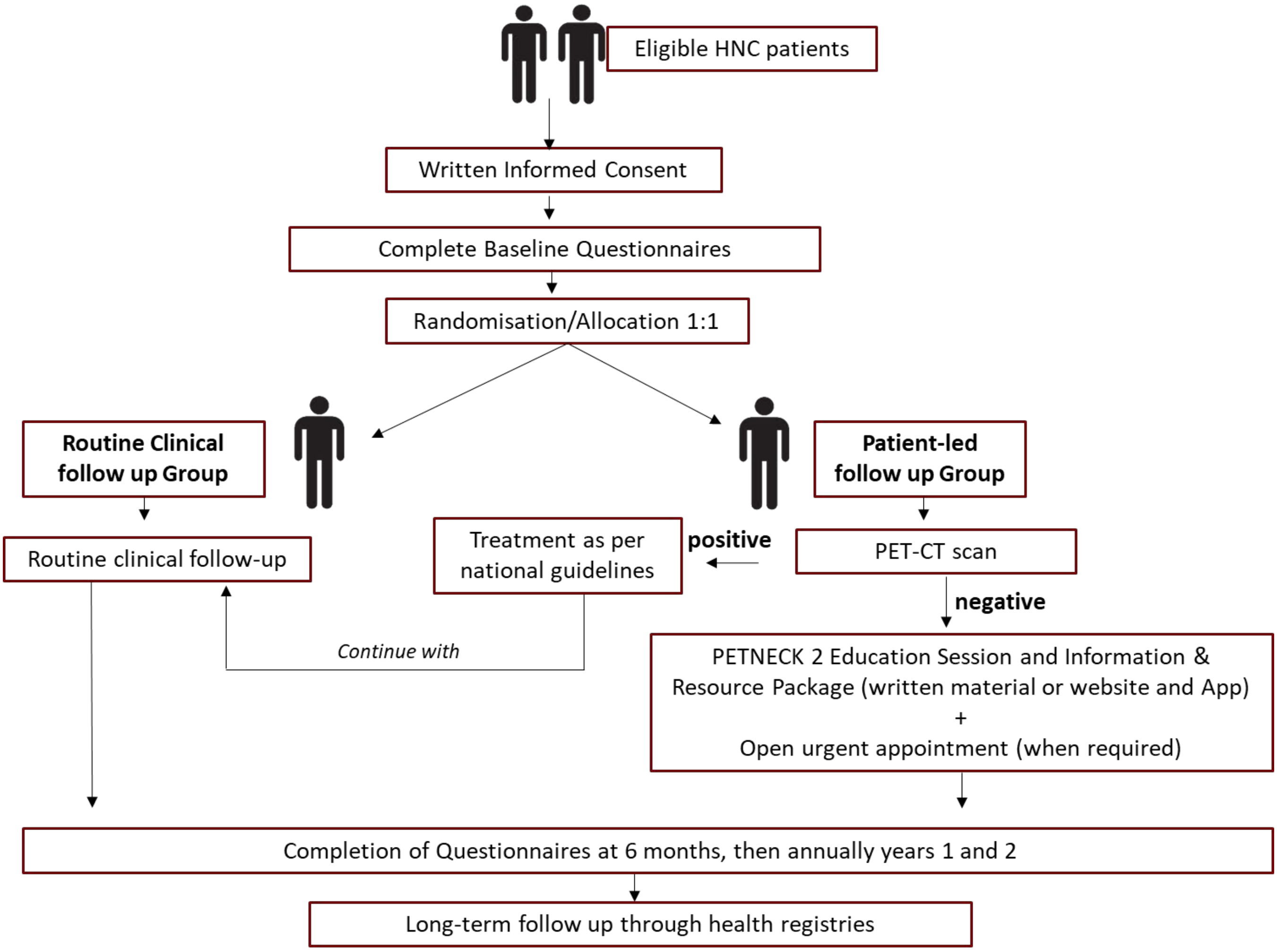

What happens if I am allocated to the group for patient-led follow-up?

If you have been allocated to the patient-led follow-up, your hospital will book you to have a PET-CT scan. *Female patients are advised to avoid pregnancy until after they have had the PET-CT scan. Once* the PET-CT scan results are available, someone from the hospital research team will contact you to discuss the results.

If the PET-CT scan is positive or unclear (a sign either of possible recurrence or cancer spread, or of something else in the body not related to your head and neck cancer), you will have further tests as recommended by UK national guidelines. If further tests confirm a recurrence, your study doctor will discuss treatment options with you. You will still be part of the trial but you will return to regular scheduled follow-up and we will collect information about your treatment as part of the study.

If the PET-CT scan is negative (no sign of recurrence or cancer spreading) or if after you have had further investigations and these rule out recurrence, you will be asked to return to the hospital for a

PETNECK2 education session with a Clinical Nurse Specialist, Speech and Language Therapist, dietician or other Allied Health Professional. This session will last approximately 20-30 minutes. Before this education session, you will receive instructions on how to download and activate the App on your mobile phone or access it via the website. You will also receive a link to watch a video that walks you through the main features of the App. PETNECK2

During the PETNECK2 education session, the following will be covered:

- Explore and discuss any concerns you might have about being on patient-led follow up
- Help you download the App, if needed
- Show you how to use the App and website, or review the paper booklet
- Discuss what is normal for you and symptoms of recurrence to look out for
- Show you how to check for any changes or new symptoms
- Tell you about the excellent additional support available from online and local support groups and charities, available through the App or paper booklet
- Add and check the contact details of your local head and neck cancer clinical team, including your key healthcare worker; tell you who to contact about concerns, and show you how to contact them
- Discuss how to contact your clinical team to make a review appointment at any time (called an ‘open urgent appointment’)
- Discuss any other general concerns you might have

Your participation in the study will continue to the end of the study , after which you will return to regular clinical follow-up. With your permission, the PETNECK2 Trial Office will continue to get information about your progress from the national health registries.

What happens if I am allocated to the group having clinic-led follow-up?

If you have been allocated to the clinic-led follow up group, your follow up will continue according to your hospital's normal practice. Your doctor will explain this to you, but it is usually every two-four months in the second year after the end of treatment, and every three to six months in years three to five.

What is a PET-CT scan?

A Positron Emission Tomography–Computed Tomography (PET-CT) scan is images taken by a type of scanner, called a PET-CT scanner, which takes pictures of the tissues and cells inside the body. Because cancer cells are much more active than other cells, the PET-CT can detect this activity. More

importantly, they are good at finding hidden cancers left behind after treatment and at detecting the absence of cancer cells in the neck. With your consent, a copy of your PET-CT scan will be held by the PETNECK2 Research Team, based at the University of Birmingham, and may be used for future research that has been approved by an ethics committee.

What is the Information Resource?

The Information Resource will be given to you before a face-to-face education session delivered by an AHP or Nurse. This resource will be an App, which can be downloaded onto your mobile phone, and/or tablet or it can also be viewed on a website. If you are not comfortable with using the App or website, you will be given a paper-based booklet. The information resource will include:

- information on key symptoms of recurrence that you should look out for;
- techniques to check yourself and reminders to check your symptoms;
- how to make an urgent appointment if you find worrying symptoms;
- how to overcome any concerns you might have about being on patient-led follow up;
- links to HNC peer support groups and cancer charities;
- links to existing information about living well after a head and neck cancer diagnosis and managing side effects;
- contact details of your head and neck cancer clinical team and other key healthcare professionals;
- a diary to for you to record your symptoms

Are there additional parts to the study?

The study also has additional parts. You can still take part in the main study even if you choose not to take part in the optional parts described below:

- **Scans - Optional:** You will be asked if you are happy for a copy of any scans you may have (CT, MRI, PET-CT and other scans) to be provided to the researchers. These will be used for future research that has been ethically and scientifically approved.
- **PETNECK2 Collect - Optional:** This will allow scientists now and in the future to study blood, tissue and oral fluid samples from patients with head and neck cancer so that they can try to understand how the cancer cells work. We also want to look at how genes in the cancer are changed. You will be asked if you want to provide blood and oral fluid samples as well as tissue from any previous surgery and pieces of any extra tissue removed surgically in future (if your cancer returns). This will be used for future research that has been approved by an ethics committee. If you agree to take part in this additional part, we will ask you to have a blood sample and a sample of oral fluid (spit from the mouth) taken at the time of study entry and at your clinic appointment and then again after one year.

- **Qualitative Interviews for Fear of Cancer Recurrence - Optional:** Fear of cancer recurrence (FCR) means ‘fear, worry, or concern about cancer returning or progressing’. We would like to understand patients’ experiences of both types of follow-up and how it affects

their fear of cancer recurrence. This part of the clinical study is optional. It will involve two interviews with PETNECK2 Researchers, one at the time of study entry and the other after a year. If you decide to participate in this part of the study, a PETNECK2 researcher will contact you to arrange a date and time suitable for you. You can choose to have the interviews either face-to-face, by telephone or video call, or by email.

- **Qualitative Recruitment Intervention (also called the 'Information Study') - Optional:** This part of the clinical study is optional. You will receive a separate information sheet describing the aims and procedures involved in this part of the study. If you agree to take part in this 'Information Study', you will be asked to sign a separate PETNECK2 Information Study Informed Consent Form.

What are the benefits to taking part?

We cannot say if being part of the study will help you or not.

Having a PET-CT scan one year after finishing treatment may have some benefits. If the scan is positive, it may detect a recurrent cancer earlier than if you did not have one and research has shown that if cancer is detected earlier there are better chances of successful treatment. If it is negative, it will provide you with some reassurance that you do not have cancer at that time.

Although you will not have regular appointments made for you with your clinical team, you will have an OPEN URGENT APPOINTMENT so that you are able to see your clinical team within 2 weeks of contacting them if any new or worrying symptoms appear. It is possible you may find not having regular appointments with your team unsettling initially as you will have been used to this in the first year after treatment. However, you may find that patient-led follow-up is more flexible and tailored to your needs, and that the less frequent clinical visits may save your time and money involved in travelling to the hospital.

Whichever group you are assigned to, you will be helping us to study the different ways of following up head and neck cancer patients, which will benefit many other future patients.

What are the possible disadvantages and risks to taking part?

If you agree to take part in the study and are selected at random to join the Group A: Patient-led follow-up arm then you will have an additional PET-CT scan. This will be extra to any other scan that you would have if you did not take part in the study. This procedure uses ionising radiation to form images of your body and provide your doctor with other clinical information. Ionising radiation may cause cancer many years or decades after the exposure. *Female patients are advised to avoid pregnancy until after they have had the PET-CT scan.*

We are all at risk of developing cancer during our lifetime. 50% of the population is likely to develop one of the many forms of cancer at some stage during our lifetime. Taking part in the Group A: Patient-led follow-up arm of the study may increase the chances of this happening to you by about 0.07%.

The scan may also detect other things in your body (not to do with your head and neck cancer) that mean you need to have further tests to find out what it is.

Blood sample collection:
If you choose to take part in ‘PETNECK2 Collect’ study, a small amount of blood will be collected by your hospital staff. This may cause temporary discomfort or you may feel faint. Sometimes a bruise or redness develops at the site where the needle was inserted. Please inform your study doctor or research nurse if you experience any reactions at the injection site.

Interview(s):
If you choose to take part in any of the interview(s), it is possible that discussing issues surrounding your cancer and follow-up care may be challenging and may cause you distress. The interview(s) will be conducted by an experienced researcher, trained in interviewing patients on potentially sensitive topics. However, if you do have any distress after taking part in an interview, we will provide contact details for support groups who may help you.

What happens when the PETNECK2 study stops?

The study will run for a period of approximately 4 years. All patients (regardless of arm) will be followed up until the last patient has had 12 months of follow up. After the end of the study, all patients in the PETNECK2 study will return to regular clinical follow-up.

With your permission, the PETNECK2 Trial Office will obtain information about your progress and health status from the national health registries.

What if there is a problem?

Any complaint about the way you have been dealt with during the study or any possible harm you might suffer will be addressed. The detailed information on this is given in Part 2.

Will my taking part in the study be kept confidential?

Yes. All the information about your participation in the study will be handled in confidence. The details are included in Part 2.

This completes Part 1.

If the information in Part 1 has interested you and you are considering participation,

please read the additional information in Part 2 before making any decision.

Part 2

Will my taking part in this study be kept confidential?

All information collected about you for this study will be subject to the EU General Data Protection Regulation and to the Data Protection Act 2018 for health and social care research and will be kept strictly confidential. All information will be securely stored at the PETNECK2 Trial Office within the Cancer Research UK Clinical Trials Unit on paper and electronically and will only be accessible by authorised personnel.

With your permission, your study doctor or research nurse will provide your name, initials, date of birth, contact details (address, email address and phone number), racial/ethnic origin, hospital and NHS number when they enter you into the study. Your study doctor will notify your GP that you intend to participate in the study. They will also send a copy of your signed Informed Consent Form with your full name and contact details to the PETNECK2 Trial Office.

In subsequent communication between your hospital and the PETNECK2 Trial Office you will only be identified by your unique trial number, initials, date of birth, hospital and NHS number. If you have consented to take part in PETNECK2 Collect sub-study, your trial number, initials and date of birth will be recorded on the blood, oral fluid and any surgical tissue samples sent to the University of Birmingham labs and the Human Biomaterials Resource Centre.

If you consent a copy of your scans (e.g. PET-CT, MRI and CT) will be pseudo-anonymised by your hospital and labelled only with your study number before they are transferred to the PETNECK2 Research Team, based at the University of Birmingham, for future ethically approved research. The original scan will be retained by your hospital. In extremely rare instances, patient identifiers may be hidden into the coding of the images dependant on the supplier of the scanning equipment. The images will be retained for future research and when the images are sought for use, they will be individually checked by the local clinical team (independent to the research team), to ensure that all identifiers are removed before any further research is conducted.

Any information that you enter into the App/website or booklet with be confidential and no one will be monitoring this. However, it would be helpful for us to understand how well patients interact with the App/website or booklet and therefore we will get information on the number of times you interact with the App/website over the period of the study.

You will need to provide your email address when activating your account for the App/website. This is to ensure that endoscope-i (App developers) can send you a confirmation of sign-up and a link to reset your password in case you forget it.

If you have consented to take part in the Information Study and/or FCR interviews, all audio/video recordings will be on encrypted devices or downloaded from Zoom iCloud and will be password protected. They will be labelled with your initials, date of birth and Trial Number and will be transferred to the Universities of Bristol and/or Stirling to be studied, where they will be stored in a locked cabinet/secure device for the duration of this study. Long term storage will be at the University of Birmingham for ten years, after which they will be erased. Only members of the research team and authorised personnel at the University of Bristol and University of Stirling will have access to the

recordings. Extracts from anonymised audio/video recordings may be played for teaching purposes or in presentations at academic meetings only if you have provided informed consent. Your name and contact details will be used to contact you to arrange the interview if you choose to take part in the interview(s).

Quotes from your interviews may also be used in publications if you have provided informed consent; however, you will not be identified. This is optional and you can choose not to allow us to do this particular aspect if you wish. All recordings will be transcribed by professional transcription companies and all transcripts will also be pseudoanonymised. This means that your name, and any personal identifiers (i.e. locations, names of hospitals or doctors) will not be included in the transcripts so that you cannot be recognised from any of the information we collect from you.

All information will be treated as strictly confidential and nothing that might identify you will be revealed to any third party other than those involved in the treatment, Qualitative Recruitment Intervention (‘Information Study’), FCR interviews or organisation of tissue, blood and oral fluid collection and transfer (e.g. staff at University of Birmingham, University of Bristol, University of Stirling, University of Oxford and Human Biomaterials Resource Centre).

By taking part in the study you will be agreeing to allow members of the research staff at your hospital and from the PETNECK2 Trial Office to look at your study records, which includes your medical records. It may also be necessary to allow the authorised personnel from government regulatory agencies (e.g. the Medicines and Healthcare products Regulatory Agency), the Sponsor and/or NHS bodies to have access to information about you. This is to ensure that the study is being conducted to the highest possible standards. If you give permission, we will also register your details with the NHS Health and Social Care Information Centre. This will enable us to collect additional long-term information.The legal justification we have under data protection law for processing your personal data is that it is necessary for our research, which is a task we carry out in the public interest. This data will not be used to make decisions about you. All individuals who have access to your information have a duty of confidentiality to you.

If you choose to withdraw from the study, we would still like to collect relevant information about your health. If you have any objection to this please let your study doctor know.

Under no circumstances will you be identified in any way in any report, presentation or publication arising from this study.

Your rights to access, change, or move your information, are limited, as we need to manage your information in specific ways in order for the research to be reliable and accurate. If you withdraw from the study, we will keep the information about you that we have already obtained. To safeguard your rights, we will use the minimum personally-identifiable information possible. Under the provisions of the EU General Data Protection Regulation, you have the right to know what information the PETNECK2 Trial Office have recorded about you. If you wish to view this information, please contact Legal Services at the address below. Please note that a small fee may be payable to retrieve this information.

Legal Services

University of Birmingham

Edgbaston

Birmingham B15 2TT

You can find out what we do with your data in our Privacy Notice see https://www.birmingham.ac.uk/crctu

If you would like more information on your rights, would like to exercise any right or have any queries relating to our processing of your personal data, please contact:

The Information Compliance Manager,

Legal Services,

University of Birmingham,

Edgbaston, Birmingham B15 2TT

Email: dataprotection@contacts.bham.ac.uk Telephone: +44 (0)121 414 3916

If you wish to make a complaint about how your data is being or has been processed, please contact our Data Protection Officer.

Nicola Cardenas Blanco,

The Data Protection Officer,

Legal Services, The University of Birmingham,

Edgbaston, Birmingham B15 2TT

Email: dataprotection@contacts.bham.ac.uk Telephone: +44 (0)121 414 3916

You also have a right to complain to the Information Commissioner's Office (ICO) about the way in which we process your personal data. You can make a complaint using the ICO’s website.

Where will my data be stored?

We will need to use information from you for this research project.

This information will include your name, initials, date of birth, hospital number, contact details (address and postcode, phone number and email address), racial/ethnic origin as well as data obtained as part of the study.

Your name, address and contact details will be shared with and transferred between the researchers at the Universities of Birmingham and Universities of Bristol, Stirling and Oxford. PETNECK2 Researchers from these organisations use this information to arrange qualitative interviews and send out research questionnaires. Researchers at Endoscope-i (App developers) will have your email address for the purpose of activating your App account.

The University of Birmingham will also share your demographic details (age, gender and ethnic background) and type and stage of your cancer with the researchers at University of Stirling, University of Bristol, Oxford Brookes and University of Oxford for the purpose of analysing the information collected from your questionnaires and interviews. If you have agreed to be interviewed for your Fear of Cancer Recurrence you may also be asked about your education and income level.

In addition the researchers at the University of Oxford will be provided with your baseline clinical data, your treatment data, clinical outcome data and with the answers to your questionnaires (i.e. the health services you used and costs).This is so that when reviewing the information they can check whether the support resource given to you is appropriate for all type of head and neck cancer, treatments, groups, ages, genders and ethnic groups. It is important that the support resource caters for all patients diagnosed with head and neck cancer.

Researchers will use this information to conduct the health economics analysis. This is a field that looks at understanding and promoting health for all.

People who do not need to know who you are will not be able to see your name or contact details. Your data will have a unique study number instead.

The data about you will be stored on secure university networks at the Universities of Birmingham, Bristol, Oxford and Stirling, in accordance with GDPR and the Data Protection Act 2018, and the Universities data management policies. We will keep all information about you safe and secure.

Long-term storage of the data will be at the University of Birmingham; the data will be stored for 10 years after the study has finished. If you withdraw from the study, we will keep the information we have already obtained but, to safeguard your rights, we will use the minimum personally-identifiable information possible.

Will my GP be involved?

It is important that your GP is kept up to date with any research study you are participating in. With your permission your GP will be informed that you are taking part in this study.

Where can you find out more about how your information is used?

You can find out more about how we use your information

- at www.hra.nhs.uk/information-about-patients/
- our leaflet available from the HRA website at www.hra.nhs.uk/patientdataandresearch
- by asking one of the research team
- by sending an email to the PETNECK2 Trial Office at PETNECK2@trials.bham.ac.uk, or
- by contacting the Sponsor's Data Protection & Freedom of Information Manager at dataprotection@contacts.bham.ac.uk

What will happen to any samples I give?

When you had your surgery for Head and Neck cancer, your cancer tissue was stored in the pathology department of your hospital. If you have agreed to take part in the PETNECK2 Collect sub-study, we will request the tissue sample from your surgery and remaining samples from any surgery you may have from your hospital pathology laboratories directly. We will also request a piece of tissue from your previous surgery and future surgeries if your cancer returns.

Your tissue samples and any blood and oral fluid samples will be identified by your trial number, initials and date of birth and will be sent for storage to the PETNECK2 Collect Laboratory and/or Human Biomaterials Resource Centre, both based at the University of Birmingham, for future research.

Tissue, blood and oral fluid samples will be stored indefinitely for future research studies at The University of Birmingham (PETNECK2 Collect Laboratory and/or the Human Biomaterials Resource Centre) in accordance with their local policy. Any future research studies will be approved by a Research Ethics Committee. We cannot describe what these future projects might involve but we will look into the way cancer develops, responds to treatment and ways in which we may predict which tumour would respond best to which treatments. This can include genetic studies looking at mechanism of action of the tumour.

Will any genetic tests be done?

We intend to study the expression of genes within the tissue and blood samples stored. In the future it may be very important to use these samples for new research on tumour genes and related areas of research.

What will happen to the results of the study?

We intend to publish the results of this research in a respected scientific or medical journal. No patients will be identified in any presentations, reports or publications resulting from the study. Patients taking part in this study can find out about the results from their study doctor once the results have been published if they have provided consent. A lay summary of the study results approved by the Research Ethics Committee will be available to you if you wish. The results will also be available on the CancerHelp website.

Who is organising and funding the research?

The research is being organised and run by the University of Birmingham in collaboration with the Universities of Bristol, Oxford, Oxford Brookes and Stirling. The study is being coordinated by the Cancer Research UK Clinical Trials Unit at The University of Birmingham.

The sponsor of the study is the University of Birmingham. This study is funded by the National Institute for Health Research (NIHR) under its Programme Grants for Applied Research Programme (project reference NIHR200861).

The views expressed are those of the author(s) and not necessarily those of the NIHR or the Department of Health and Social Care.

Expenses and payments

You will not receive any money for taking part in this study. If you have consented to take part in the FCR interview, we will reimburse travel and parking expenses to attend the interview, if it takes place outside of your home.

Your study doctor, research nurse or Allied Health Professional will not receive any payments for including you in this study.

Who has reviewed the study?

All research in the NHS is looked at by an independent group of people, called a Research Ethics Committee, to protect your safety, rights, wellbeing and dignity. This study has been reviewed and given favourable opinion by a Research Ethics Committee. It has also been reviewed by the Research and Development Office at your hospital.

What if there is a problem?

Complaints

In an unlikely event of you having a concern about any aspect of this research study, you should contact your study doctor in the first instance. You can use the contact number at the end of this sheet.

If you remain unhappy and wish to complain formally, you can do this through the Patient Advice and Liaison Services (PALS) or the National Health Service (NHS) complaints procedure. Details can be obtained from your hospital.

Harm

In the event that something does go wrong and you are harmed and this is due to someone's negligence then you may have grounds for legal action for compensation against the sponsor of the study (University of Birmingham) or the NHS Trust but you may have to pay your legal costs, as would be the situation even if you were not in a study. NHS Trust and Non-Trust Hospitals have a duty of care to patients treated, whether or not the patient is taking part in a study and the normal NHS

complaints mechanisms will still be available to you. The Sponsor of the study does not hold insurance against claims for compensation for injury caused by participation in this study and they cannot offer any indemnity.

Further Information and Contact Details

What happens now?

You will have some time to think about the study and make your decision. You may wish to discuss it with your family or friends. If you take part, you will receive a copy of this information sheet and a copy of the signed consent form to take home. Your doctor will inform your GP of your decision to take part in the study. If, at any time, you have any questions about the study you should contact your study doctor or research nurse using the details below.

Local contact details:

Study Doctor: ____

Research Nurse: ____

☐: ____

Emergency (24 hours)

☐: ____

You may also find it helpful to contact the following organisations:

Your local Patient Advice and Liaison Service (PALS) or local equivalent who provide advice and support to patients, their families and their carers; website: http://www.nhs.uk

The contact number of your local Patient Advice and Liaison Service or local equivalent is:

[Delete as appropriate]Should you have any complaints you can contact the Patient Advice and Liaison Services (PALS) or the National Health Service (NHS). Your local PALS is:

[Enter local details]

[Delete as appropriate] In Northern Ireland the Patient Client Council (PCC) can provide assistance and support at any stage of the health and social care services complaints procedure. The PCC is an independent body who represent the views of the public in all areas of health and social care. They can also assist you to make a complaint. This is a confidential and free service.

http://www.patientclientcouncil.hscni.net/

Telephone: 0800 917 0222

Email: info.pcc@hscni.net

[Delete as appropriate] The Patient Advice and Support Service is an independent service which provides free, accessible and confidential information, advice and support to patients, their carers, and families about NHS healthcare in Scotland.

http://www.patientadvicescotland.org.uk/

[Delete as appropriate] Community Health Councils (CHCs) are independent bodies, set up by law, who listen to what individuals and the community have to say about the health services with regard to quality, quantity, access to and appropriateness of the services provided for them. CHCs can also help, advise and support people who wish to make complaints about NHS services and similar matters. This advice is completely free, independent and confidential.

http://www.wales.nhs.uk/sitesplus/899/page/99722

CancerHelp, an information service about cancer from Cancer Research UK, Freephone 0808 800 40 40, website: www.cancerhelp.org.uk

Macmillan Cancer Support: Freephone 0808 800 0000, website: www.macmillan.org.uk

Thank you for taking the time to read this information sheet.
